# Supplementary material for: Qualitative study to identify ethnicity-specific perceptions of and barriers to asthma management in South Asian and White British children with asthma
Source: BMJ Open. 2019 Feb 8;9(2):e024545. doi: 10.1136/bmjopen-2018-024545 (PMC6411253; doi:10.1136/bmjopen-2018-024545)
Supplement: Supplementary file 1 [file bmjopen-2018-024545supp001.pdf]

Supplementary file 1: Semi-structured interview question schedule

Question schedule for children

Hello. Thank you very much for agreeing to speak to me. So I'm (interviewer), and this is an interview for the MIA research study. We' re going to talk about your breathing. I'm going to ask you things like what you remember from when you were very little, and I'm going to ask you if you remember what it's like now. I'll also ask some questions about what happens when you have a bad day, medicines you take and what other people say about your asthma. If you don't know the answer, you can just guess. There's no right or wrong answer.

1. When did your breathing problems first start?
    - Initial symptoms/recognition
    - Who/how diagnosed
    - Information/support provided
    - Barriers to diagnosis
        - Seeking help
        - HCP attitude
        - Access to service
2. People have lots of different Ideas about why some children get wheezy. Why do you think experience wheeziness?
    - Triggers vs. cause
    - Heritability
    - Contagion
    - Gods will
    - Diet

3. What do you think causes asthma/ wheezing?
4. Could you describe what's your asthma like on a day-to-day basis
    - Symptoms
    - Impact on day-to-day life
    - Lifestyle restriction
    - Time off work
    - Family life
    - Schooling
5. Who makes the decision about day-to-day things?
    - Mum/dad
    - Child
    - Extended family
6. Could you describe a recent asthma attack? (substitute bad attack if little response)
    - Describe management
        - Use of health services
        - Use of tests/diagnostics
        - Role of extended family in management
7. Problems faces
    - Recognition of need to seek help
    - Access to service A&E/ walk in/ GP
    - Practicalities- carrying medicines, time of day, weekends etc
    - HCP communication/attitudes/ethnicity/ gender
8. What worked well?
    - Good service

- Good person

9. Information provided
    - What
    - From who
    - Useful/not
    - What else wanted
    - Timing
10. Solutions used/wanted
11. Let's talk about treatments. What do you hope treatments will do for your asthma/wheezing?
    - Personal goals of treatment
    - Management vs. cure
    - Concept of asthma control
    - What is good control
12. Do you take any medicines for your asthma/wheezing? (substitute inhalers if no response) (go through medicine one by one)
    - When do you use this/how do you feel about it/any problems getting taking it/ how do you remember to take it/ does everyone in the family know about it/ use in public?
    - For preventer medicines: when do you stop/ who makes decisions/ side effects?
    - Specific attitudes
        - Long term med use
        - Inhaler vs. tablets
        - Steroids

13. Who has helped you learn about wheezing/asthma?
    - Sources of knowledge
    - Skills
    - Overseas advice
    - Role of schools/community centres/religious centres
    - Use of written management plans
14. What do the rest of the family think about your asthma?
    - Who knows/ gets involved
    - What do you think
    - Does anyone offer advice/ do you follow it?
    - Impact on management
15. What about your friends?
    - Do you tell others/ stigma?
    - Would you tell about another illness/specific to asthma?
    - Common beliefs about cause
    - Common attitudes towards asthma/ children with asthma
    - Advice offered/ follow it
    - Has anyone upset you?
    - Do cultural attitudes influence management?
16. Where do you go for help about your asthma?
    - Useful source of care- GP/A&E/walk-in/family and friends/internet/religious leaders
        - Who do you go to?
        - What is the key issue- opening hours, location, relationship, gender, ethnicity

        - Problems faced getting help
            - Communication with HCPs
            - Getting prescriptions

17. What would overcome these?

18. Is there anything that you've found particularly helpful in taking care of your asthma?

    - What/why?
    - Alternative therapies used?
        - What/why
        - Diet
        - Heating/furnishing changes
        - Barriers or facilitator to using different options

19. Is there anything else we could do to help you cope with your asthma?

    - What/why/how
        - Specific to your family
        - General suggestions for families and children
