# Supplementary material for: Qualitative study to identify ethnicity-specific perceptions of and barriers to asthma management in South Asian and White British children with asthma
Source: BMJ Open. 2019 Feb 8;9(2):e024545. doi: 10.1136/bmjopen-2018-024545 (PMC6411253; doi:10.1136/bmjopen-2018-024545)
Supplement: Author's manuscript [file bmjopen-2018-024545.draft_revisions.pdf]

BMJ Open

BMJ Open is committed to open peer review. As part of this commitment we make the peer review history of every article we publish publicly available.

When an article is published we post the peer reviewers' comments and the authors' responses online. We also post the versions of the paper that were used during peer review. These are the versions that the peer review comments apply to.

The versions of the paper that follow are the versions that were submitted during the peer review process. They are not the versions of record or the final published versions. They should not be cited or distributed as the published version of this manuscript.

BMJ Open is an open access journal and the full, final, typeset and author-corrected version of record of the manuscript is available on our site with no access controls, subscription charges or pay-per-view fees (http://bmjopen.bmj.com).

If you have any questions on BMJ Open's open peer review process please email info.bmjopen@bmj.com

A qualitative study to identify ethnic-specific perceptions of and barriers to asthma management in South Asian and White British children with asthma

Journal:	*BMJ Open*
Manuscript ID	bmjopen-2018-024545
Article Type:	Research
Date Submitted by the Author:	31-May-2018
Complete List of Authors:	Lakhanpaul, Monica; University College London, Institute of Child Health; Whittington Health NHS Trust, Paediatrics Department<br>Culley, Lorraine; De Montfort University, School of Applied Social Sciences<br>Huq, Tausif; King's College London, School of Medical Education<br>Bird, Deborah ; London North West Healthcare NHS Trust<br>Hudson, Nicky; DeMontfort University, School of Applied Social Science<br>Robertson, Noelle ; University of Leicester Centre for Medical Humanities, Clinical Psychology<br>McFeeters, Melaine ; NHS England , Specialised Commissioning East Midlands<br>Manikam, Logan; UCL Institute of Child Health, Population, Policy & Practice<br>Johal , Narynder ; Parent representative<br>Hamlyn-Williams , Charlotte ; University College London, Population, Policy and Practice, Institute of Child Health<br>Johnson, Mark; DeMonfort University, Mary Seacole Research Centre
Keywords:	Asthma < THORACIC MEDICINE, Community child health < PAEDIATRICS, QUALITATIVE RESEARCH, PAEDIATRICS, PUBLIC HEALTH

SCHOLARONE™
Manuscripts

A qualitative study to identify ethnic-specific perceptions of and barriers to asthma management in South Asian and White British children with asthma

Corresponding author

1. **Professor Monica Lakhanpaul**

Institutional address: Population, Policy and Practice, UCL Great Ormond Street Institute of Child Health, 30 Guildford Street, London WC1N 1EH, UK

Email address: m.lakhanpaul@ucl.ac.uk

Telephone number: +44 (0)20 7905 2259

Consultant Paediatrician

Whittington Health NHS Trust

2. Professor Lorraine Culley

Institutional address: School of Applied Social Sciences, De Montfort University, The Gateway, Leicester, LE1 9BH, UK

Email address: lac@dmu.ac.uk

Telephone number: +44 (0)116 257 7753

3. Tausif Huq

Institutional address: King's College London, Guy's King's and St Thomas' Hospital (GKT) School of Medicine, New Hunt's House, Guy's Campus, London, SE1 1UL, UK

Email address: tausif.huq@kcl.ac.uk

For peer review only

Telephone number: +44 (0)7454003467

4. Dr Deborah Bird

Institutional address: Ealing Child Development Team, Carmelita House, 21-22 The Mall, Ealing, W5 2PL

Email address: deborah.bird@nhs.net

Telephone number: +44 (0) 208 825 8700

5. Dr Nicky Hudson

Institutional address: School of Applied Social Sciences. De Montfort, University, The Gateway, Leicester, LE1 9BH, UK

Email address: nhudson@dmu.ac.uk

Telephone number: +44 (0)116 207 8766

6. Dr Noelle Robertson

Institutional address: Clinical Psychology, University of Leicester, Centre for Medicine, Lancaster Road, Leicester, LE1 7HA, UK

Email address: nr6@le.ac.uk

Telephone number: +44 (0)116 223 1617/1648

7. Dr Melanie McFeeters

Institutional address: Specialised Commissioning East Midlands, NHS England, Fosse House, 6 Smith Way, Grove Park, Enderby, Leicestershire, LE19 1SX, UK

Email address: Melanie.Mcfeeters@nhs.net

For peer review only

Telephone number: +44 (0) 7717 720185

8. Dr Logan Manikam

Institutional address: Department of Epidemiology & Public Health, UCL Institute of Epidemiology & Healthcare, 1-19 Torrington Place, London WC1E 7HB, UK

Email address: logan.manikam.10@ucl.ac.uk

Telephone number: +44 (0)7868721152

9. Narynder Johal

Institutional address: Parent representative, Leicester, UK

Email address: narynder@hotmail.com

Telephone number: n/a

10. Dr Charlotte Hamlyn-Williams

Institutional address: Population, Policy and Practice, UCL Great Ormond Street Institute of Child Health, 30 Guildford Street, London WC1N 1EH, UK

Email address: c.hwilliams@outlook.com

Telephone number: +44 (0)7748282923

11. Professor Mark R D Johnson

Institutional address: Mary Seacole Research Centre, De Montfort University, The Gateway, Leicester, LE1 9BH, UK

Email address: mrdj@dmu.ac.uk

Telephone number: +44 (1) 676 532389

For peer review only

Abstract

Objective: Current research indicates that minority ethnic groups, especially South Asian children in the UK encounter excessive healthcare inequalities, particularly in managing chronic conditions such as asthma. This includes higher morbidity and hospitalisation rates compared to the White British population. This paper draws on the data from the Management and Interventions for Asthma (MIA) study to explore the perceptions and experiences of asthma in British South Asian children by semi-structured interview. A comparable cohort of White British children were recruited to identify if the emerging themes were subject to variation between the two groups so that generic and ethnic-specific themes can be identified for future tailored intervention programmes for South Asian children with asthma.

Setting: South Asian and White British children with asthma took part in semi-structured interviews in Leicester.

Participants: 33 South Asian and 14 White British children with asthma aged 5-12 years were interviewed.

Results: Both similar and contrasting themes emerged from the semi-structured interviews. Interviews revealed considerable similarities in the experience of asthma between the South Asian and White British children including the lack of understanding of asthma (often confusing trigger with cause), lack of holistic discussions with healthcare professionals (HCPs), an overall neutral or positive experience of interactions with HCPs, the role of family in children's self-management and the positive role of school and friends. Issues pertinent to South Asian children related to the higher likelihood of feeling embarrassed and attributing

For peer review only

physical activity as trigger of asthma symptoms.

Conclusions: The two ethnic-specific factors revealed by the semi-structured interviews are vital in children's self-management of asthma and therefore indicate the need for a tailored intervention in South Asian children.

Keywords: Asthma, Paediatrics, Qualitative Research, Collaborative research Community child health, Public health

Word count: Abstract- 272 words, Text- 3000 words, Table- 1

Article summary

Strength and limitation of this study:

- An 'Expression of interest' form enabled children and parents from previous phases of the MIA study to participate in subsequent phases and thus achieve a continuous community consultative process.

- An unequal power relationship between an adult researcher and child participant has been mitigated through interviews being carried out by two female paediatricians trained in qualitative methods

- The study was not resourced to analyse children's drawings, although if done systematically, this may provide additional useful insight into children's understanding and experience of asthma.

- Some children declined to participate in the activities provided, such as drawing, and the activities themselves increased the length of interview time.

For peer review only

Introduction

Asthma is one of the most prevalent chronic conditions of childhood. Estimates in the UK suggests 1.1 million, or one in ten children will experience asthma at some point in their childhood[1]. Evidence shows that children from South Asian communities suffer from under-recognition of asthma symptoms, disease severity and increased attendance at the emergency department, exceeding their White British counterparts[2–4]. However, there is no evidence to indicate South Asian children experience more severe asthma or have a genetic predisposition to severe asthma attacks.[2–4]

Barriers to effective management of asthma in South Asian children are well recognised in the literature[2–4]. However, many health interventions aimed at mitigating these barriers have failed to improve asthma outcomes, often ascribed to a lack of cultural sensitivity and a failure to recognise the ethno-religious heterogeneity in the South Asian community[5]. A systematic review by Lakhanpaul *et al*. identified several cultural and structural barriers to optimal asthma management in South Asian children, including diverse knowledge of asthma, non-acceptance and denial of asthma by parents[6]. It highlighted the importance of identifying the nature and mechanism of these barriers so that ethnically-tailored interventions can be developed. Unlike 'modified' interventions, which modify an intervention originally developed for a majority population, 'tailored' interventions take into account individual cultural influences within a group, and therefore are more effective[6,7].

For peer review only

There is also a remarkable under-representation of UK ethnic minority children in all aspects of asthma research, thus specific ethnic-specific and religious-specific factors are not integrated into health services[8]. This is despite international (*UN Convention on the Rights of the Child*[9]) and national policy mandates (including the Children Act 1989 and 2004 affecting England and Wales) to increase precedence of the views and opinions of children and young people (CYP). As CYP are not a homogenous group, and have varying degrees of health needs depending on backgrounds, capabilities and interests, it is important that their views are heard, especially in childhood asthma.

Context of the current study: the MIA project

This paper is drawn from the Management and Intervention for Asthma (MIA) study-carried out in an urban area in Leicester, UK. It had an overall aim to develop an intervention-planning framework for the British South Asian children with asthma, guided by a participatory collaborative method which involved individuals and communities defining problems and developing solutions[10].

This paper reports on one of the major phases of the MIA study: to explore the perceptions and experiences of asthma in British South Asian children by semi-structured interviews. A comparable cohort of White British children helped to identify if the emerging themes were subject to variation between the two groups, so that generic and ethnic-specific themes can be identified for future tailored intervention programmes in British South Asian children with asthma. Children's interviews were complemented by interviews with parents/carers, community members and health care professionals (HCPs), the results of which are discussed elsewhere[10].

For peer review only

Methods

This study adopted a qualitative methodology using semi-structured interviews.

Participant sampling

Families with children aged 5-12 years with a diagnosis of asthma were included (Table 1). Participants were purposively sampled via two routes: via health care (e.g. general practice) and the community, facilitated by community facilitators.

Recruitment ensured a proportional representation of six major South Asian ethno-religious groups within one geographical area (Table 1, these terms describe ethno-religious identity and not participant's nationality), with parents asked to assign children's ethnicity and demographic information. From the recruited families (saturation target n=30), further purposive sampling was used to recruit children (matching for age, sex and asthma severity according to British Thoracic Society guidelines[11]).

Thirty-three South Asian children and 14 White children were interviewed. BTS grading allowed a wide range of asthma experiences to be captured (Table 1).

For peer review only

Consent and assent

An 'Expression of interest' form enabled both children and parents from previous phases of the MIA study to participate in subsequent phases and thus achieve a continuous community consultative process.

Adults (parents/legal guardians) consented on behalf of their children. Assessment of 'Gillick competency' was used to obtain consent from children aged 10-12-years[12]. Younger children were judged by the research team on their ability to provide assent orally where appropriate.

Data collection

The question schedule was developed from the findings of an initial scoping review and by external advisors (Supplementary file 1)[10]. Children also produced drawings and text which guided interview questions. Interviews were carried out by two female research fellows (qualified paediatrician, trained in qualitative methods and not involved in their care). Interviews typically lasted between 60-90 minutes with no repeat interviews conducted. Interviews took place in the children's family home (some chose to give interviews with parents) and were digitally recorded and transcribed. Field notes were taken during each interview.

Data analysis

Interview transcripts were analysed according to the principles of interpretive thematic analysis using NVivo (QSR International, Warrington, UK)[13]. Transcripts were not returned to children for comments/corrections.

A detailed explanation of the thematic analysis can be found elsewhere[10,14]. Briefly, two analysts independently analysed the transcripts to create a framework of

For peer review only

thematic categories from both interviews. The thematic frameworks of South Asian and White British children's data were then closely compared and amended to ensure consistency of meaning across both datasets to create a comprehensive coding framework. All transcripts were then systematically coded using the resultant framework.

Ethics

Ethical approval was gained through the local NHS ethics committee (ref. 09/H0401/85), University ethics and NHS R&D approval. All participants provided written or verbal consent.[10]

For peer review only

Results

The key themes arising from the exploration of the interviews were understandings of asthma, children's self-management of asthma, perception of asthma, management in school, the role of friends, and interactions with healthcare providers.

Children's understandings of asthma

Fourteen South Asian children (42%) and six White British children (43%) explained what they thought to be the reasons for asthma, although most were uncertain. Some children attributed causes of asthma to triggers such as the weather and the environment, or viruses.

"Well I think it's where if you are near something that triggers it off your lungs might tighten together a little bit and stop you breathing and lock up your air pipe a little bit so you will get quite wheezy" (White British boy)

"As mum keeps saying over and over again, the damp weather, […] I may have caught asthma from the damp weather…". (Indian girl)

Only two South Asian and two White British children expressed some insights into the hereditary component of asthma.

"I know it runs in families and stuff because me and my mum have got it". (White British girl)

For peer review only

"Probably because like someone in their family before like your grandma or grandfather had it [...]" (Punjabi boy)

Children's interactions with healthcare system

Seven South Asian (21%) and seven White British children (50%) could not recall the time they were diagnosed with asthma, which may reflect the quality of interaction they had had with the HCPs. Children were more likely to remember their recent interactions, suggesting a lack of recall may be age-and time-dependent.

Overall, children's interactions with the HCPs were broadly of a positive or neutral nature. Children were more likely to recall their visits to their general practitioner (GP) since most had visited recently. Positive comments focused on the GP's friendly approach and communications, including explaining asthma and medications. Three negative comments related to "horrible tasting tablets", misdiagnosis of hay fever and being "ignored" after initiating nebuliser treatment.

"He [doctor], he'll tell you what's happening, what's wrong, and if you have asthma or if you don't have asthma, and he'll tell you if you need a, the stuff for the inhaler you when you put it in." (Pakistani boy)

In both groups, experiences and perceptions of secondary care were generally negative, often involving aversive emotional descriptions and related to being treated for acute asthma attacks. Nevertheless, the quality of the interaction with individual HCPs remained positive.

"Just a little bit scary. […] Because sometimes you have to have injections. [The nurses are] so kind. […] So what [the doctors] do injections they, when they've done it, [the nurses] cheer me up when I cry." (Indian girl)

For peer review only

South Asian and White British children did not recall having a holistic discussion with the HCPs such as discussions about school, physical activity and quality of life. Some also recalled GPs not involving children during consultations.

"[Doctors should talk to] both [me and my parents][…] Like, to explain […] what is it happening, […] after explaining to the parents[…]" (Bangladeshi girl)

Children's self-management of asthma

Twenty nine South Asian (89%) and 4 White British children (29%) spoke about self-assessing symptoms and self-management, especially as they became older. In both groups, parents were responsible for reminding and instructing children to take inhalers and facilitating the process of self-management.

The families' response to an asthma attack appeared to mediate a child's symptom perception. In both groups, children reported that parents, especially their mothers, played a vital role in managing acute attacks by spotting symptoms and encouraging inhaler use.

"[…] I had to literally run to get my pump and . . . I couldn't breathe properly. So I had to take one [inhaler] […] But sometimes it might be mum, she can go 'oh [name] I would take your pump if I were you" (Indian girl)

It was evident that the older children devised their own system of self-management, with the help of their parents.

"…so always have two inhalers of the same colour, like… if this one ran out then I'd tell my Mum or Dad or sister…" (Indian boy)

For peer review only

Overall, parents (especially mothers) play an important role in mediating children's symptom perception and self-management of routine and acute attacks in both groups of children with no remarkable ethnic-specific beliefs identified.

Children's perception of the impact of asthma

One of the biggest impacts of asthma as reported by the children affected their ability to participate in sporting activities and physical education classes. South Asian children were more likely to attribute physical activity as a trigger to an asthma attack (n=29, 89%) than White British children (n=7, 50%). Common symptoms included breathlessness and coughing with children either sitting out or avoiding activities altogether.

"Because you know when I run the wheeziness starts and make me cough […] So that' s why I take the inhaler sometimes when we come back from sports or anything." (Indian boy)

"I have had to do it once [take inhaler] before but it was sports day […]." (*White British girl*)

The most commonly reported emotions were embarrassment (n=11 South Asian; n=3 White British), fear (n=3 South Asian; n=2 White British), upset (n=7 South Asian; n=1 White British) and unfairness (n=2 South Asian; n=2 White British). South Asian children were more likely to report embarrassment in relation to sports, school and taking medication than White British children. It was described however with some emotional distance as an embarrassing condition rather than children being embarrassed (Further details in *School and friends*).

"[…] she's grown up, she says, It's embarrassing to carry it [inhaler], mum. " (Mother

For peer review only

of a Bangladeshi girl)

"That I have to cough when my sister doesn't cough, or I have to have more medication than she has to have. It's a bit annoying sometimes but you get used to it." (White British girl)

Both groups of children expressed few positive emotions and when asked if they had one wish, many reported that they wanted asthma to 'go away'. A few children did find some positives in their experience of asthma management.

"The good thing is erm […] I think I've improved in my confidence and my determination […]." (Bangladeshi boy)

School and friends

Peers and friends were described as having an important role in children's experience and management of asthma. Having friends who understood their asthma, and in some cases, could help if needed, was deemed important for both South Asian and White British children.

"At school it doesn't affect me much, people don't tease me or anything… , their friends are aware of my asthma, some of them have asthma themselves and they help me". (Pakistani boy)

"… they [friends] mostly help me, like they usually go get it [inhaler] for me." (White British girl)

Asthma was considered a cause to feel different or 'not normal', thus a small number of children described strategies to manage the amount of information they disclosed

For peer review only

to their peers.

"I have to explain it a lot . . . they understand I have got it and I think they are alright because they know it's not catching." (White British girl)

Some children reported that only their close friends knew about their condition. One White British girl felt that using her inhaler at school attracted unwanted attention and one South Asian child reported being teased about his asthma.

Suggestions for service improvements

Some children along with their parents discussed ways in which knowledge of asthma could be improved and offered ideas for future interventions. Children reported wanting to speak to someone who had experience of asthma, either living with asthma or an HCP. Children and families also described ways in which information could be delivered including use of pictures, involving school and teachers, places of worship and accessible/reliable information online.

"Pictures maybe." (Indian boy)

"A presentation[. . .]. They tell you things that I did not know like in more detail.[. . .] The chemist and the GP were ok but the school one explained it best and easy to understand." (Indian boy)

"They [school] could do a little show about asthma or something like that […]" (White British girl)

For peer review only

Discussion

The Children's Outcome Forum report recommends considering strategies to work closely with children to improve health outcomes[15]. Therefore, the semi-structured interviews with children in the MIA study provided an opportunity for those affected by asthma to offer their experiences and perceptions of their asthma management. It revealed many of the problems facing these children are common to all communities. However, key ethnic-specific experiences relevant to South Asian children are also highlighted: feelings of embarrassment and attributing physical activity to asthma symptom triggers.

Children as young as 6-years-old are able to self-manage asthma with parents having a positive intermediary effect. Therefore, knowledge of asthma will support and nurture the practice of self-management. However, both groups of children expressed confusions and misconceptions of the causes, triggers, and nature of asthma[16,17]. These findings indicate that the HCPs may not be adequately delivering health information and/or checking the understanding of those receiving the information. The need for an improved information provision is also echoed by the children by offering suggestions on the various methods of delivery.

Interactions with HCPs were mostly reported as positive or neutral by the South Asian and White British children and not reveal any ethnic-specific barriers to communication. In contrast, semi-structured interviews with the children's parents/carers revealed language and literacy barriers as a factor to effective communication associated with limited knowledge of asthma. Some children described being 'left out' during doctors' consultations, which were often directed at

For peer review only

their parents. Children often recalled providing a clinical history and being examined but did not recollect having any holistic discussions. Indeed, this is consistent with data collected from an earlier phase of the MIA study[18] which found that HCPs tended to focus on addressing the medications with limited discussions about holistic management.

Physical training is actively recommended within asthma management guidelines[19]. Our results show that South Asian children are more likely to attribute physical activity as trigger of asthma symptoms than the White British children. Indeed, it has been reported that South Asian children are 3.6 times more likely to suffer from exercise-induced bronchoconstriction than White British children[20]. Regardless of the underlying mechanism, our results indicate the need for a tailored approach to change the perceptions of physical activity in South Asian children.

In the interviews, a desire to be normal was commonly expressed by the children, echoing previous studies examining the psychosocial elements of asthma[21,22]. The children struggled to talk positively about asthma, with negative emotions equally apparent in both groups. However, the self-conscious emotion of embarrassment was more likely to be experienced by the South Asian children, and often related to day-to-day activities such as sports, or medication, when the children were seen as visibly different, rather than having asthma per se. Children who feel embarrassed are less likely to carry or use inhalers with an overall negative impact on self-management[21]. A sense of embarrassment can be further compounded with the indirect experience of asthma-related stigmatisation ("courtesy stigma"[23]), as South Asian families may experience negative reactions from the wider South Asian community[10,14]. Therefore, a tailored intervention to tackle emotional affects in South

For peer review only

Asian children should be considered.

Challenges and limitations

Semi-structured interviews with young children can create an unequal power relation between an adult researcher and child participant[24,25]. However, a broad mix of positive and negative experiences suggest this to be minimal. Furthermore, interviewing young children also increases costs, resources such as toys, time implication and in some instances loss of interest in involvement (n=2).

Policy Practice and Conclusion

Interviews with children from South Asian and White British background revealed considerable similarities in the experiences and perceptions of asthma management. It thus appears important to work with both South Asian and White British children to design an intervention programme, which includes the issues raised in this qualitative study. However, the two ethnic-specific themes emerged from the interviews suggest that there is also a need for a more tailored intervention in South Asian children. Additionally, the MIA study also interviewed parents/carers, HCPs and community members and revealed a significant variation in the barriers and facilitators to asthma management in South Asian children, which can indirectly affect children's experience[10]. Together with the data from this study and the previous MIA studies, it can contribute to the development of a multifaceted tailored intervention to improve the management of asthma in South Asian children.

For peer review only

Abbreviations

HCPs: Health Care Professionals; GPs: General Practitioners; PE: Physical education

Declarations

Data sharing statement

No additional data are available

Ethics approval and consent to participate

Ethical approval was gained through the local NHS ethics committee (ref. 09/H0401/85), University ethics and NHS R&D approval. All participants gave written or verbal consent to participate in the study.

Competing interests

During the MIA project, Monica Lakhanpaul was appointed as a National Institute for Health and Care Excellence (NICE) Fellow, member of the NHS Evidence advisory board, the Health Technology Assessment advisory panel and the Drugs and Therapeutics Bulletin editorial panel.

Funding

This project was funded by the National Institute for Health Research. Health Services and Delivery Research (HS&DR) programme (project number 09/2001/19). NIHR had no role in the design, analysis or writing of this article.

Logan Manikam is funded by a National Institute for Health Research (NIHR) Doctoral

For peer review only

Research Fellowship (DRF-2014-07-005). Monica Lakhanpaul is funded by the NIHR Collaboration for Leadership in Applied Health Research and Care (CLAHRC) North Thames at Bart's Health NHS Trust.

Authors' contributions

ML, DB, LC, NR, NK, NR, NJ, MM, MJ made substantial contributions to the conception and design of the study. ML was responsible for the overall direction of the project. ML, NR, DB, LC, NH, NR, NJ, MM, EA, CM, MJ contributed to the analysis and interpretation of data. ML, DB, LC, NR, NK, NR, NJ, MM, MJ contributed to the systematic evidence synthesis. ML, DB, LC, NR, NK, NR, NJ, MM designed and delivered the participatory workshops. ML, DB, LC, NR, NH, NR, NJ, MM, CM, LM, TH, MJ contributed to the drafting of the report and have given final approval of this report.

Acknowledgements

The authors would like to express their thanks to the following: All families, children, community members and healthcare professionals who participated. The community facilitators and advisory committees.* Professor Jonathan Grigg who contributed to the conception and design of the study and gave advice throughout the project. External advisor, Professor Mike Thomas. Maya Lakhanpaul, Chelsea Huddlestone and Aadil Ali who were our youth facilitators. Ms Gill Perkins and Jo Wilson who assisted with the reviewing of the literature for the systematic evidence synthesis in phase 1. Mrs Stephanie Langan, Mrs Elaine Huddlestone and Mrs Liz Knight. Mrs Emma L. Angell for coding and summarising interview data. Ms Emma Alexander for her thoughtful comments on the draft manuscript. University of Leicester, Leicester Royal Infirmary and the Children's Assessment Unit, Leicester Community Child Health Services, all participating community centres, Mosques

For peer review only

453 and Gurdwaras, organisations, creches*, LCEHR. Asthma UK, with particular thanks to
454 Brigid Hall and Leanne Metcalf. The PCRN and CLRN and the Leicester Community
455 Children's Partnership. Dr Jo Forster. Dr Neena Lakhani. Dr Marian Carey. De Montfort
456 University. Staff at our recruitment sites.*

457 This research was supported by the National Institute for Health Research (NIHR)
458 Collaboration for Leadership in Applied Health Research and Care North Thames at Bart's
459 Health NHS Trust (NIHR CLAHRC North Thames). The views expressed in this article are
460 those of the author(s) and not necessarily those of the NHS, the NIHR, or the Department of
461 Health and Social Care.

462

463 *Refer to published report for further details

For peer review only

References:

1. Asthma UK. Asthma facts and statistics. at <https://www.asthma.org.uk/about/media/facts-and-statistics/>

2. Netuveli, G. *et al.* Ethnic variations in UK asthma frequency, morbidity, and health-service use: a systematic review and meta-analysis. *Lancet* **365,** 312–317 (2005).

3. Asthma UK. The asthma divide : inequalities in emergency care for people with asthma in England. - Abstract - Europe PMC. *2012*

4. Kuehni, C. E., Strippoli, M.-P. F., Low, N., Brooke, A. M. & Silverman, M. Wheeze and asthma prevalence and related health-service use in white and south Asian pre-schoolchildren in the United Kingdom. *Clin. Exp. Allergy* **37,** 1738–1746 (2007).

5. Ahmed, S., Steed, L., Harris, K., Taylor, S. J. C. & Pinnock, H. Interventions to enhance the adoption of asthma self-management behaviour in the South Asian and African American population: a systematic review. *npj Prim. Care Respir. Med.* **28,** 5 (2018).

6. Lakhanpaul, M. *et al.* A systematic review of explanatory factors of barriers and facilitators to improving asthma management in South Asian children. *BMC Public Health* **14,** 403 (2014).

7. Bailey, E., Kruske, S., Morris, P., Cates, C. & Chang, A. Culture-specific programs for children and adults from minority groups who have asthma. *Cochrane Database Syst. Rev.* CD006580 (2008). doi:10.1002/14651858.CD006580.pub2

8. Hussain-Gambles, M. *et al.* Involving South Asian patients in clinical trials. *Health Technol. Assess.* **8,** iii, 1-109 (2004).

9. UNICEF. *Convention on the Rights of the Child*. (2010).

10. Lakhanpaul, M. *et al. The use of a collaborative structured methodology for the development of a multifaceted intervention programme for the management of asthma*

For peer review only

(the MIA project), tailored to the needs of children and families of South Asian origin: a community-based, partici. (NIHR Journals Library, 2014). doi:10.3310/HSDR02280

11. THE British Thoracic Society. SIGN 141- British guideline on the management of asthma. (2014).

12. Griffith, R. What is Gillick competence? *Hum. Vaccin. Immunother.* **12,** 244–7 (2016).

13. Silverman, D. *Interpreting Qualitative Data: Methods for Analysing Talk, Text and Interaction*. (2001).

14. Lakhanpaul, M. *et al.* A qualitative study to identify parents' perceptions of and barriers to asthma management in children from South Asian and White British families. *BMC Pulm. Med.* **17,** 126 (2017).

15. Lewis, I., Lenehan, C. & Shribman, S. *Report of the Children and Young People's Health Outcomes Forum*. (2012).

16. PALMER, L. J. *et al.* Independent Inheritance of Serum Immunoglobulin E Concentrations and Airway Responsiveness. *Am. J. Respir. Crit. Care Med.* **161,** 1836–1843 (2000).

17. Duffy, D. L., Martin, N. G., Battistutta, D., Hopper, J. L. & Mathews, J. D. Genetics of Asthma and Hay Fever in Australian Twins. *Am. Rev. Respir. Dis.* **142,** 1351–1358 (1990).

18. Lakhanpaul, M. *et al.* The use of a collaborative structured methodology for the development of a multifaceted intervention programme for the management of asthma (the MIA project), tailored to the needs of children and families of South Asian origin: a community-based, participatory study. *Heal. Serv. Deliv. Res.* **2,** 1–226 (2014).

19. British Thoracic Society & Scottish Intercollegiate Guidelines Network. *British guideline on the management of asthma*. (2016).

20. Jones, C. O., Qureshi, S., Rona, R. J. & Chinn, S. Exercise-induced

For peer review only

515 bronchoconstriction by ethnicity and presence of asthma in British nine year olds. *Thorax* **51,** 1134–6 (1996).

517 21. Cohen, R., Franco, K., Motlow, F., Reznik, M. & Ozuah, P. O. Perceptions and attitudes of adolescents with asthma. *J. Asthma* **40,** 207–11 (2003).

519 22. Callery, P., Milnes, L., Verduyn, C. & Couriel, J. Qualitative study of young people's and parents' beliefs about childhood asthma. *Br. J. Gen. Pract.* **53,** 185–90 (2003).

521 23. Macrae, H. Managing courtesy stigma: the case of Alzheimer's disease. *Sociol. Health Illn.* **21,** 141–9889 (1999).

523 24. Punch, S. Research with Children: The Same or Different from Research with Adults? *Childhood* **9,** 321–341 (2002).

525 25. Gardner, H. & Randall, D. The effects of the presence or absence of parents on interviews with children. *Nurse Res.* **19,** 6–10 (2012).

Table 1: Demographic information of semi-structured interview participants

	South Asian children (%)	White British children (%)
Total number	33	14
Male	20 (61)	8 (57)
Female	13 (39)	6 (43)
BTS grading		
Level 1	7 (21)	3 (21)
Level 2	17 (52)	8 (57)
Level 3	6 (18)	3 (21)
Level 4	3 (9)	0
Ethno-religious/ethnic group		
Indian Gujarati Hindu	6 (18)	-
Indian Gujarati Muslim	4 (12)	-
Indian Gujarati Jain	1 (3)	-
Indian Punjabi Sikh	6 (18)	-
Indian Punjabi Hindu	1 (3)	-

For peer review only

**Bangladeshi Muslim**	**9 (27)**	**-**
**Pakistani Muslim**	**6 (18)**	**-**
**White British**	**-**	**13 (93)**
**White British (English)**		**1 (7)**
**White British (Wales)**	**-**	**0**
**White British (Scottish)**	**-**	**0**
**White British (Northern Irish)**	**-**	**0**
**Religion**		
**Hindu**	**7 (21)**	**0**
**Muslim**	**19 (58)**	**0**
**Sikh**	**6 (18)**	**0**
**Jain**	**1 (3)**	**0**
**Christian**	**0**	**6 (43)**
**Atheist**	**0**	**1 (7)**
**No religious belief**	**0**	**4 (29)**
**Not specified**	**0**	**3 (21)**

For peer review only

**Age**		
**5 years old**	**2 (6)**	**2 (14)**
**6 years old**	**5 (15)**	**2 (14)**
**7 years old**	**5 (15)**	**0**
**8 years old**	**4 (12)**	**4 (29)**
**9 years old**	**3 (9)**	**1 (7)**
**10 years old**	**5 (15)**	**4 (29)**
**11 years old**	**5 (15)**	**1 (7)**
**12 years old**	**3 (9)**	**0**
**Not specified**	**1 (3)**	**0**
**Parental/ carer highest level of education achieved ***		
**Primary school**	**4 (8)**	**0**
**Secondary school**	**21 (43)**	**11 (37)**
**College education**	**3 (6)**	**3 (10)**
**University**	**20 (41)**	**14 (47)**
**Not specified**	**1 (2)**	**2 (7)**

530

*** Numbers may not equal to the total number of children interviewed as either one or both parents responded to the questionnaire**

For peer review only

Supplementary file 1: Semi-structured interview question schedule

Question schedule for children

Hello. Thank you very much for agreeing to speak to me. So I'm (interviewer), and this is an interview for the MIA research study. We' re going to talk about your breathing. I'm going to ask you things like what you remember from when you were very little, and I'm going to ask you if you remember what it's like now. I'll also ask some questions about what happens when you have a bad day, medicines you take and what other people say about your asthma. If you don't know the answer, you can just guess. There's no right or wrong answer.

1. When did your breathing problems first start?
    - Initial symptoms/recognition
    - Who/how diagnosed
    - Information/support provided
    - Barriers to diagnosis
        - Seeking help
        - HCP attitude
        - Access to service
2. People have lots of different Ideas about why some children get wheezy. Why do you think experience wheeziness?
    - Triggers vs. cause
    - Heritability
    - Contagion
    - Gods will
    - Diet

For peer review only

3. What do you think causes asthma/ wheezing?
4. Could you describe what's your asthma like on a day-to-day basis
    - Symptoms
    - Impact on day-to-day life
    - Lifestyle restriction
    - Time off work
    - Family life
    - Schooling
5. Who makes the decision about day-to-day things?
    - Mum/dad
    - Child
    - Extended family
6. Could you describe a recent asthma attack? (substitute bad attack if little response)
    - Describe management
        - Use of health services
        - Use of tests/diagnostics
        - Role of extended family in management
7. Problems faces
    - Recognition of need to seek help
    - Access to service A&E/ walk in/ GP
    - Practicalities- carrying medicines, time of day, weekends etc
    - HCP communication/attitudes/ethnicity/ gender
8. What worked well?
    - Good service

For peer review only

- Good person

9. Information provided
    - What
    - From who
    - Useful/not
    - What else wanted
    - Timing
10. Solutions used/wanted
11. Let's talk about treatments. What do you hope treatments will do for your asthma/wheezing?
    - Personal goals of treatment
    - Management vs. cure
    - Concept of asthma control
    - What is good control
12. Do you take any medicines for your asthma/wheezing? (substitute inhalers if no response) (go through medicine one by one)
    - When do you use this/how do you feel about it/any problems getting taking it/ how do you remember to take it/ does everyone in the family know about it/ use in public?
    - For preventer medicines: when do you stop/ who makes decisions/ side effects?
    - Specific attitudes
        - Long term med use
        - Inhaler vs. tablets
        - Steroids

For peer review only

13. Who has helped you learn about wheezing/asthma?
    - Sources of knowledge
    - Skills
    - Overseas advice
    - Role of schools/community centres/religious centres
    - Use of written management plans
14. What do the rest of the family think about your asthma?
    - Who knows/ gets involved
    - What do you think
    - Does anyone offer advice/ do you follow it?
    - Impact on management
15. What about your friends?
    - Do you tell others/ stigma?
    - Would you tell about another illness/specific to asthma?
    - Common beliefs about cause
    - Common attitudes towards asthma/ children with asthma
    - Advice offered/ follow it
    - Has anyone upset you?
    - Do cultural attitudes influence management?
16. Where do you go for help about your asthma?
    - Useful source of care- GP/A&E/walk-in/family and friends/internet/religious leaders
        - Who do you go to?
        - What is the key issue- opening hours, location, relationship, gender, ethnicity

For peer review only

- Problems faced getting help
  - Communication with HCPs
  - Getting prescriptions

17. What would overcome these?

18. Is there anything that you've found particularly helpful in taking care of your asthma?

- What/why?
- Alternative therapies used?
  - What/why
  - Diet
  - Heating/furnishing changes
  - Barriers or facilitator to using different options

19. Is there anything else we could do to help you cope with your asthma?

- What/why/how
  - Specific to your family
  - General suggestions for families and children

For peer review only

Consolidated criteria for reporting qualitative studies (COREQ): 32-item checklist[1]

No	Item	Guide questions/description	Checklist
**Domain 1: Research team and reflexivity**			
**Personal Characteristics**			
**1.**	Interviewer/facilitator	Which author/s conducted the interview or focus group?	Dr Deborah Bird
**2.**	Credentials	What were the researcher's credentials? *E.g. PhD, MD*	See 'authors and institution' Line 4-71
**3.**	Occupation	What was their occupation at the time of the study?	See 'authors and institution' Line 4-71
**4.**	Gender	Was the researcher male or female?	Female Line 194
**5.**	Experience and training	What experience or training did the researcher have?	Line 195-196
**Relationship with participants**			
**6.**	Relationship established	Was a relationship established prior to study commencement?	Yes; via encouraging children to draw and write about their asthma. Line 193-194
**7.**	Participant knowledge of the interviewer	What did the participants know about the researcher? e.g. *personal goals, reasons for doing the research*	Supplementary file 1
**8.**	Interviewer characteristics	What characteristics were reported about the interviewer/facilitator? e.g. *Bias, assumptions, reasons and interests in the research topic*	Line 194-196. Interviewer characteristics reported are: gender, profession, level of training in research and conflict of

			interests
**Domain 2: study design**			
**Theoretical framework**			
**9.**	Methodological orientation and Theory	What methodological orientation was stated to underpin the study? *e.g. grounded theory, discourse analysis, ethnography, phenomenology, content analysis*	Semi-structured interview (grounded theory) Line 164
**Participant selection**			
**10.**	Sampling	How were participants selected? *e.g. purposive, convenience, consecutive, snowball*	Purposive sampling Line 173-179
**11.**	Method of approach	How were participants approached? e.*g. face-to-face, telephone, mail, email*	Face-to-face and telephone Line 171-172
**12.**	Sample size	How many participants were in the study?	Line 180
**13.**	Non-participation	How many people refused to participate or dropped out? Reasons?	Due to loss of interest, some children did not wish to participate in the interviews (n=2). Line 398
**Setting**			
**14.**	Setting of data collection	Where was the data collected? e.*g. home, clinic, workplace*	Family home. Line 197
**15.**	Presence of non-participants	Was anyone else present besides the participants and researchers?	Some independently and some with parents. Line 198
**16.**	Description of sample	What are the important characteristics of the sample? *e.g. demographic data, date*	Table 1. Line 530-533
**Data collection**			
**17.**	Interview guide	Were questions, prompts, guides provided by the	Supplementary file 1

		authors? Was it pilot tested?	
**18.**	Repeat interviews	Were repeat interviews carried out? If yes, how many?	No repeat interviews were conducted. Line 196-197
**19.**	Audio/visual recording	Did the research use audio or visual recording to collect the data?	Interviews were digitally voice-recorded. Line 198
**20.**	Field notes	Were field notes made during and/or after the interview or focus group?	Fields notes were taken. Line 199
**21.**	Duration	What was the duration of the interviews or focus group?	Interviews typically lasted 60-90 minutes Line 196
**22.**	Data saturation	Was data saturation discussed?	Line 165177
**23.**	Transcripts returned	Were transcripts returned to participants for comment and/or correction?	Transcripts were not returned to children for comments/ corrections. Line 202-203
**Domain 3: analysis and findings**			
**Data analysis**			
**24.**	Number of data coders	How many data coders coded the data?	There were two data coders. Line 205
**25.**	Description of the coding tree	Did authors provide a description of the coding tree?	Line 193-199
**26.**	Derivation of themes	Were themes identified in advance or derived from the data?	Open coding. Line 204-210
**27.**	Software	What software, if applicable, was used to manage the data?	NVivo. Line 202
**28.**	Participant checking	Did participants provide feedback on the findings?	Transcripts were not returned to

			children for comments/ corrections. Line 202-203
**Reporting**			
**29.**	Quotations presented	Were participant quotations presented to illustrate the themes / findings? Was each quotation identified? e.g. *participant number*	Results Line 216-343
**30.**	Data and findings consistent	Was there consistency between the data presented and the findings?	Discussion. Line 345-411
**31.**	Clarity of major themes	Were major themes clearly presented in the findings?	Discussion. Line 345-411
**32.**	Clarity of minor themes	Is there a description of diverse cases or discussion of minor themes?	Discussion. Line 345-411

1. Allison Tong, Peter Sainsbury, Jonathan Craig; Consolidated criteria for reporting qualitative research (COREQ): a 32-item checklist for interviews and focus groups, *International Journal for Quality in Health Care*, Volume 19, Issue 6, 1 December 2007, Pages 349–357, https://doi.org/10.1093/intqhc/mzm042

For peer review only

A qualitative study to identify ethnic-specific perceptions of and barriers to asthma management in South Asian and White British children with asthma

Journal:	*BMJ Open*
Manuscript ID	bmjopen-2018-024545.R1
Article Type:	Research
Date Submitted by the Author:	28-Aug-2018
Complete List of Authors:	Lakhanpaul, Monica; University College London Institute of Child Health, Population, Policy and Practice ; Whittington Health NHS Trust, Department of Paediatrics<br>Culley, Lorraine; De Montfort University, School of Applied Social Sciences<br>Huq, Tausif; King's College London, School of Medical Education<br>Bird, Deborah ; London North West Healthcare NHS Trust, Ealing Child Development Team<br>Hudson, Nicky; DeMontfort University, School of Applied Social Science<br>Robertson, Noelle ; University of Leicester Centre for Medical Humanities, Clinical Psychology<br>McFeeters, Melaine ; NHS England , Specialised Commissioning East Midlands<br>Manikam, Logan; University College London Institute of Epidemiology and Health Care, Department of Epidemiology & Public Health<br>Johal , Narynder ; Parent representative<br>Hamlyn-Williams , Charlotte ; University College London Institute of Child Health, Population, Policy and Practice<br>Johnson, Mark; DeMonfort University, Mary Seacole Research Centre
**Primary Subject Heading**:	Paediatrics
Secondary Subject Heading:	Respiratory medicine, Public health, Health services research, Qualitative research
Keywords:	Asthma < THORACIC MEDICINE, Community child health < PAEDIATRICS, QUALITATIVE RESEARCH, PAEDIATRICS, PUBLIC HEALTH

SCHOLARONE™
Manuscripts

A qualitative study to identify ethnic-specific perceptions of and barriers to asthma management in South Asian and White British children with asthma

Corresponding author

1. **Professor Monica Lakhanpaul**

Institutional address: Population, Policy and Practice, UCL Great Ormond Street Institute of Child Health, 30 Guildford Street, London WC1N 1EH, UK

Email address: m.lakhanpaul@ucl.ac.uk

Telephone number: +44 (0)20 7905 2259

Professor of Integrated Community Child Health

Consultant Paediatrician

Whittington Health NHS Trust

2. Professor Lorraine Culley

Institutional address: School of Applied Social Sciences, De Montfort University, The Gateway, Leicester, LE1 9BH, UK

Email address: lac@dmu.ac.uk

Telephone number: +44 (0)116 257 7753

3. Tausif Huq

Institutional address: King's College London, Guy's King's and St Thomas' Hospital (GKT) School of Medicine, New Hunt's House, Guy's Campus, London, SE1 1UL, UK

For peer review only

Email address: tausif.huq@kcl.ac.uk

Telephone number: +44 (0)7454003467

4. Dr Deborah Bird

Institutional address: Ealing Child Development Team, Carmelita House, 21-22 The Mall, Ealing, W5 2PL

Email address: deborah.bird@nhs.net

Telephone number: +44 (0) 208 825 8700

5. Prof. Nicky Hudson

Institutional address: School of Applied Social Sciences. De Montfort, University, The Gateway, Leicester, LE1 9BH, UK

Email address: nhudson@dmu.ac.uk

Telephone number: +44 (0)116 207 8766

6. Prof. Noelle Robertson

Institutional address: Clinical Psychology, University of Leicester, Centre for Medicine, Lancaster Road, Leicester, LE1 7HA, UK

Email address: nr6@le.ac.uk

Telephone number: +44 (0)116 223 1617/1648

7. Dr Melanie McFeeters

Institutional address: Specialised Commissioning East Midlands, NHS England, Fosse House, 6 Smith Way, Grove Park, Enderby, Leicestershire, LE19 1SX, UK

For peer review only

Email address: Melanie.Mcfeeters@nhs.net

Telephone number: +44 (0) 7717 720185

8. Dr Logan Manikam

Institutional address: Department of Epidemiology & Public Health, UCL Institute of Epidemiology & Healthcare, 1-19 Torrington Place, London WC1E 7HB, UK

Email address: logan.manikam.10@ucl.ac.uk

Telephone number: +44 (0)7868721152

9. Narynder Johal

Institutional address: Parent representative, Leicester, UK

Email address: narynder@hotmail.com

Telephone number: n/a

10. Dr Charlotte Hamlyn-Williams

Institutional address: Population, Policy and Practice, UCL Great Ormond Street Institute of Child Health, 30 Guildford Street, London WC1N 1EH, UK

Email address: c.hwilliams@outlook.com

Telephone number: +44 (0)7748282923

11. Professor Mark R D Johnson

Institutional address: Mary Seacole Research Centre, De Montfort University, The Gateway, Leicester, LE1 9BH, UK

Email address: mrdj@dmu.ac.uk

For peer review only

Telephone number: +44 (1) 676 532389

Abstract

Objective: This paper draws on the data from the Management and Interventions for Asthma (MIA) study to explore the perceptions and experiences of asthma in British South Asian children using semi-structured interviews. A comparable cohort of White British children were recruited to identify whether emerging themes were subject to variation between the two groups so that generic and ethnic-specific themes can be identified for future tailored intervention programmes for South Asian children with asthma.

Setting: South Asian and White British children with asthma took part in semi-structured interviews in Leicester, UK.

Participants: 33 South Asian and 14 White British children with asthma aged 5-12 years were interviewed.

Results: Both similar and contrasting themes emerged from the semi-structured interviews. Interviews revealed considerable similarities in the experience of asthma between the South Asian and White British children, including the lack of understanding of asthma (often confusing trigger with cause), lack of holistic discussions with healthcare professionals (HCPs), an overall neutral or positive experience of interactions with HCPs, the role of family in children's self-management and the positive role of school and friends. Issues pertinent to South Asian children related to the higher likelihood of feeling embarrassed and attributing physical activity as trigger of asthma symptoms.

For peer review only

Conclusions: The two ethnic-specific factors revealed by the semi-structured interviews are significant in children's self-management of asthma and therefore indicate the need for a tailored intervention in South Asian children.

Keywords: Asthma, Paediatrics, Qualitative Research, Collaborative research Community child health, Public health

Word count: Abstract- 233 words, Text- 2999 words, Table- 1

Article summary

Strengths and limitations of this study:

- An unequal power relationship between an adult researcher and child participant has been mitigated through the use of 'child-friendly' data collection methods.
- The study was not resourced to analyse children's drawings, although if done systematically, this may provide additional useful insight into children's understanding and experience of asthma.
- Some children declined to participate in the activities provided, such as drawing, and the activities themselves increased the length of interview time.

For peer review only

Introduction

Asthma is one of the most prevalent chronic conditions in childhood. Estimates in the UK suggests 1.1 million, or one in ten children will experience asthma at some point in their childhood[1]. Evidence shows that children from South Asian communities experience under-recognition of asthma symptoms, worse disease severity and increased attendance to emergency department, exceeding the rates of their White British counterparts[2–4]. However, there is no evidence to indicate South Asian children experience more severe asthma or have a genetic predisposition to severe asthma attacks.[2–4]

Barriers to effective asthma management in South Asian children are well recognised in the literature[2–4]. However, many health interventions aimed at mitigating these barriers have failed to improve asthma outcomes, often ascribed to a lack of cultural sensitivity and a failure to recognise the ethno-religious heterogeneity in the South Asian community[5]. A systematic review by Lakhanpaul *et al*. identified several cultural and structural barriers to optimal asthma management in South Asian children, including diverse knowledge of asthma, non-acceptance and parental denial of asthma[6]. It highlighted the importance of identifying the nature and mechanism of these barriers so that ethnically-tailored interventions can be developed. ‘Tailored’ interventions consider individual cultural influences, and are more effective than ‘modified’ interventions, which modify an intervention designed for a majority population[6,7].

For peer review only - http://bmjopen.bmj.com/site/about/guidelines.xhtml

There is also a remarkable under-representation of UK minority ethnic children in all aspects of asthma research, thus specific ethnic-specific and religious-specific factors are rarely integrated into health services[8]. This is despite international (*UN Convention on the Rights of the Child*[9]) and national policy mandates (including the Children Act 1989 and 2004 affecting England and Wales) to increase precedence of the views and opinions of children and young people (CYP). As CYP are not a homogenous group, and have varying health needs depending on backgrounds, capabilities and interests, it is important that their views are heard, especially in childhood asthma.

Context of the current study: the MIA project

This paper is drawn from the Management and Intervention for Asthma (MIA) study- carried out in an urban area in Leicester, UK. It had an overall aim to develop an intervention-planning framework for British South Asian children with asthma, guided by a participatory collaborative method which involved individuals and communities defining problems and developing solutions[10].

This paper reports on one of the major phases of the MIA study: to explore the perceptions and experiences of asthma in British South Asian children by semi-structured interviews. A comparable cohort of White British children were recruited to identify whether emerging themes were subject to variation between the two groups, so that generic and ethnic-specific themes could be identified for future tailored intervention programmes in British South Asian children with asthma. Children's interviews were complemented by interviews with parents/carers, community members and healthcare professionals (HCPs), the results of which are discussed elsewhere[10].

For peer review only - http://bmjopen.bmj.com/site/about/guidelines.xhtml

For peer review only

Methods

This study adopted a qualitative methodology using semi-structured interviews.

Participant sampling

Families with children aged 5-12 years with a diagnosis of asthma were included (Table 1). Participants were purposively sampled via two routes: via healthcare (e.g. general practice) and the community, facilitated by community facilitators (CFs), who were bilingual members of the South Asian community, trained as lay researchers to assist with research.

Recruitment ensured a proportional representation of six major South Asian ethno-religious groups (Table 1, these terms describe ethno-religious identity and not participants' nationality), with parents asked to assign children's demographic information. From the recruited families, purposive sampling was used to recruit children (matching age, sex and asthma severity according to British Thoracic Society guidelines[10]). In some instances, more than one child was recruited from a family.

Thirty-three South Asian children and 14 White children were interviewed. BTS grading allowed a wide range of asthma experiences to be captured (Table 1).

For peer review only

Consent and assent

Adults (parents/legal guardians) consented on behalf of their children. Assessment of 'Gillick competency' was used to obtain consent from children aged 10-12-years[11]. Younger children were judged by the research team on their ability to provide assent orally where appropriate.

Data collection

A question schedule was developed from the findings of an initial scoping review and by external advisors (Supplementary file 1)[10]. Children also produced drawings and text during the interviews which guided interview questions. Interviews were carried out by two female researchers (a paediatrician and a social scientist, both trained in qualitative methods and not involved in their care). Interviews took place in children's family homes with some choosing to give interviews with their parents. Interviews were digitally recorded, transcribed and field notes taken. No repeat interviews were conducted.

Data analysis

Interview transcripts were analysed according to the principles of interpretive thematic analysis using NVivo (QSR International, Warrington, UK)[10]. Transcripts were not returned to children for comments/corrections.

A detailed explanation of the thematic analysis can be found in previous publications[10,12]. Briefly, two analysts independently analysed the transcripts to create a framework of thematic categories from both interviews. The thematic frameworks of South Asian and White British children's data were then closely compared and amended to ensure consistency of meaning across both datasets to

For peer review only - http://bmjopen.bmj.com/site/about/guidelines.xhtml

create a comprehensive coding framework. All transcripts were then systematically coded using the resultant framework.

Patient and public involvement

Children were not directly involved in the design, recruitment or conduct of the study but participated in the development of an intervention framework following the interviews, in a series community-based workshop[10]. Parents were included as members of the research team (NJ, as co-applicant) and as members of the advisory group. Finally, a number of dissemination events were held in the community involving South Asian and White British children.

For peer review only

For peer review only - http://bmjopen.bmj.com/site/about/guidelines.xhtml

Results

The key themes arising from the exploration of the interviews were: understandings of asthma; children's self-management of asthma; perception of asthma; management in school; the role of friends; and interactions with healthcare providers.

Children's understandings of asthma

Fourteen South Asian children (42%) and six White British children (43%) explained what they thought to be the reasons for asthma, although most were uncertain. Some children attributed causes of asthma to triggers such as the weather, the environment, or viruses.

"Well I think it's where if you are near something that triggers it off your lungs might tighten together a little bit and stop you breathing and lock up your air pipe a little bit so you will get quite wheezy" (White British boy)

"As mum keeps saying over and over again, the damp weather, […] I may have caught asthma from the damp weather…". (Indian girl)

Two South Asian and two White British children thought that asthma had a hereditary component.

"I know it runs in families and stuff because me and my mum have got it". (White British girl)

"Probably because like someone in their family before like your grandma or

For peer review only - http://bmjopen.bmj.com/site/about/guidelines.xhtml

grandfather had it [...]" (Punjabi boy)

Children's interactions with healthcare system

Seven South Asian (21%) and seven White British children (50%) could not recall the time they were diagnosed with asthma. Furthermore, children were more likely to remember their recent interactions with HCPs rather than those in the past.

Overall, children's interactions with HCPs were described broadly as positive or neutral. Children were more likely to recall their visits to their general practitioner (GP) since most had visited recently. Positive comments focused on GP's friendly approach and communications, including explaining asthma and medications. Three negative comments related to "horrible tasting tablets", misdiagnosis of hay fever and being "ignored" after initiating nebuliser.

"He [doctor], he'll tell you what's happening, what's wrong, and if you have asthma or if you don't have asthma, and he'll tell you if you need a, the stuff for the inhaler you when you put it in." (Pakistani boy)

In both groups, experiences and perceptions of secondary care were generally less than positive, often involving negative emotional descriptions and related to being treated for acute asthma attacks. Nevertheless, the quality of interactions with individual HCPs remained positive.

"Just a little bit scary. […] Because sometimes you have to have injections. [The nurses are] so kind. […] So what [the doctors] do injections they, when they've done it, [the nurses] cheer me up when I cry." (Indian girl)

South Asian and White British children did not recall having a holistic discussion with

For peer review only

the HCPs such as discussions about school, physical activity and quality of life. Some also recalled GPs not involving children during consultations.

"[Doctors should talk to] both [me and my parents][…] Like, to explain […] what is it happening, […] after explaining to the parents[…]" (Bangladeshi girl)

Children's self-management of asthma

Twenty-nine South Asian (89%) and four White British children (29%) spoke about self-assessing symptoms and self-management, especially as they became older. In both groups, parents were described as being responsible for reminding and instructing children to take inhalers and facilitating the process of self-management.

The families' response to an asthma attack appeared to mediate a child's symptom perception. Both groups of children reported that parents, especially their mothers, played a vital role in managing acute attacks by spotting symptoms and encouraging inhaler use.

"My mum tells me...how many puffs...when she wants to do something else, […]she...she...she says how many times to do it. And then I start doing it. […]Sometimes my dad helps me when I'm like stuck. (Indian girl)

It was evident that the older children devised their own system of self-management, with the help of their parents.

"…so always have two inhalers of the same colour, like… if this one ran out then I'd tell my Mum or Dad or sister…" (Indian boy)

Overall, parents (especially mothers) were felt to play an important role in mediating

For peer review only

For peer review only - http://bmjopen.bmj.com/site/about/guidelines.xhtml

children's symptom perception and self-management of routine and acute attacks in both groups of children with specific ethnic differences identified.

Children's perception of the impact of asthma

One of the biggest impacts of asthma reported by the children was how it affected their ability to participate in sporting activities and physical education classes at school. South Asian children were more likely to attribute physical activity as a trigger for an asthma attack (n=29, 89%) than White British children (n=7, 50%). Common symptoms included breathlessness and coughing, resulting in children either sitting out or avoiding activities altogether.

"Because you know when I run the wheeziness starts and make me cough […] So that' s why I take the inhaler sometimes when we come back from sports or anything." (Indian boy)

"I have had to do it once [take inhaler] before but it was sports day […]." (*White British girl*)

The most commonly reported emotions were embarrassment (n=11 South Asian; n=3 White British), fear (n=3 South Asian; n=2 White British), upset (n=7 South Asian; n=1 White British) and unfairness (n=2 South Asian; n=2 White British). South Asian children were more likely to report embarrassment in relation to sports, school and taking medication than White British children. It was described however with some emotional distance as an embarrassing condition rather than children being embarrassed (See *School and friends*).

"[…] she's grown up, she says, It's embarrassing to carry it [inhaler], mum. " (Mother of a Bangladeshi girl)

For peer review only

"That I have to cough when my sister doesn't cough, or I have to have more medication than she has to have. It's a bit annoying sometimes but you get used to it." (White British girl)

Both groups of children expressed few positive emotions and when asked if they had one wish, many reported that they wanted asthma to 'go away'. A few children did find some positives in their experience of asthma management.

"The good thing is erm […] I think I've improved in my confidence and my determination […]." (Bangladeshi boy)

School and friends

Peers and friends were described as having an important role in children's experience and management of asthma. Having friends who understood their asthma, and in some cases, could help if needed, was deemed important for both South Asian and White British children.

"At school it doesn't affect me much, people don't tease me or anything… , their friends are aware of my asthma, some of them have asthma themselves and they help me". (Pakistani boy)

"… they [friends] mostly help me, like they usually go get it [inhaler] for me." (White British girl)

Asthma was considered a reason to feel different or 'not normal', thus a small number of children described strategies to manage the amount of information they disclosed to their peers.

For peer review only

For peer review only - http://bmjopen.bmj.com/site/about/guidelines.xhtml

"I have to explain it a lot . . . they understand I have got it and I think they are alright because they know it's not catching." (White British girl)

One White British girl felt that using inhaler at school attracted unwanted attention and one South Asian child reported being teased about his asthma.

Suggestions for service improvements

Some children discussed ways in which knowledge of asthma could be improved and offered ideas for future interventions. Children reported wanting to speak to someone who had experience of asthma, either living with asthma or an HCP. Children also described ways in which information can be delivered.

"Pictures maybe." (Indian boy)

"A presentation[. . .]. They tell you things that I did not know like in more detail.[. . .] The chemist and the GP were ok but the school one explained it best and easy to understand." (Indian boy)

"They [school] could do a little show about asthma or something like that […]" (White British girl)

For peer review only

Discussion

Semi-structured interviews with children in this study provided an opportunity for those affected by asthma to offer their experiences and perceptions of asthma management. The interviews revealed that some challenges perceived as facing children with asthma may be common regardless of ethnicity. However, certain experiences appeared to be more relevant to South Asian children, in particular feelings of embarrassment and attributing physical activity as a trigger for asthma symptoms.

Children as young as 6-years-old are able to self-manage asthma with parents having a positive intermediary effect[13]. Therefore, knowledge of asthma will support and nurture the practice of self-management. However, both groups of children expressed confusions and misconceptions about the causes, triggers, and nature of asthma[14,15]. These findings indicate that the HCPs may not be adequately delivering health information and/or checking the understanding of those receiving the information. The need for improved information provision is echoed by the children in the suggestions they offer for improved asthma support.

Interactions with HCPs were mostly reported as positive or neutral by the South Asian and White British children and did not reveal any ethnic-specific barriers to communication. Some children described being ‘left out’ during doctors’ consultations, which were often directed at their parents. Children often recalled providing a clinical history and being examined but did not recollect having any holistic discussions. However, this lack of recall of interactions with HCPs may relate to children’s age and the time of their recent interaction. Furthermore, this is

For peer review only - http://bmjopen.bmj.com/site/about/guidelines.xhtml

consistent with data collected from an earlier phase of the MIA study[10] which found that HCPs tended to focus on addressing medications with limited discussions about holistic management.

Physical activity is recommended within asthma management guidelines[16]. Our results show that South Asian children are more likely to attribute physical activity as a trigger for asthma symptoms than the White British children. Indeed, it has been reported that South Asian children are 3.6 times more likely to suffer from exercise-induced bronchoconstriction than White British children[17]. Regardless of the underlying mechanism, our results may indicate the need for a tailored approach to change the perceptions of physical activity in South Asian children.

A desire to be normal was commonly expressed by the children, echoing previous studies examining the psychosocial elements of asthma[18,19]. The children struggled to talk positively about asthma, with negative emotions equally apparent in both groups. However, the self-conscious emotion of embarrassment was more likely to be experienced by the South Asian children, and often related to day-to-day activities such as sports, or medication, when the children were seen as visibly different, rather than having asthma per se. However, this must be interpreted cautiously as there were more 'older children' (11-12-year olds) in South Asian cohort compared to White British children, and this perception may be a reflection of the age difference. Nevertheless, the sense of embarrassment may be compounded by an indirect experience of asthma-related stigmatisation ("courtesy stigma"[20]) from the wider South Asian community[10,12]. Children who feel embarrassed are less likely to carry or use inhalers with an overall negative impact on self-management[18]. Therefore, a tailored intervention to tackle emotional affects in South Asian children should be

For peer review only

considered.

Challenges and limitations

Semi-structured interviews with children can involve an unequal power relation between an adult researcher and child participant with differing power dynamics at play when interviewed with or without parents[21,22]. Efforts were made to minimize the impact of this, using child-friendly strategies[10]. The children's age range (5-12 years) is also a limiting factor on the data, potentially influencing asthma experience and recall. Furthermore, interviewing young children also increases costs, both in terms of resources such as toys and increased time needed to establish trust and maintain interest.

Policy Practice and Conclusion

Interviews with children from South Asian and White British communities revealed considerable similarities in the experiences and perceptions of asthma and its management. It is therefore important to work with both South Asian and White British children to design interventions which take into consideration the important common issues raised in this qualitative study. However, two ethnic-specific themes emerged from the interviews, which suggest that there may also be a need for an intervention to include a more tailored component for South Asian children, particularly around physical activity and social stigma. The MIA study also included interviews and focus groups with parents/carers, HCPs and wider community members which revealed further ethnic specific barriers and facilitators to asthma management in South Asian children, which can indirectly affect children's experience[10]. These data can contribute to the development of a multifaceted

For peer review only

For peer review only - http://bmjopen.bmj.com/site/about/guidelines.xhtml

407 tailored intervention to improve the management of asthma in South Asian children.

For peer review only

409 **Abbreviations**

410 HCPs: Healthcare Professionals; GPs: General Practitioners; PE: Physical education

411 **Declarations**

412 **Data sharing statement**

413 No additional data are available

414 **Ethics approval and consent to participate**

415 Ethical approval was gained through the local NHS ethics committee (ref. 09/H0401/85),
416 University ethics and NHS R&D approval. All participants gave written or verbal consent to
417 participate in the study.

418 **Competing interests**

419 During the MIA project, Monica Lakhanpaul was appointed as a National Institute for Health
420 and Care Excellence (NICE) Fellow, member of the NHS Evidence advisory board, the
421 Health Technology Assessment advisory panel and the Drugs and Therapeutics Bulletin
422 editorial panel.

423 **Funding**

424 This project was funded by the National Institute for Health Research. Health Services and
425 Delivery Research (HS&DR) programme (project number 09/2001/19). NIHR had no role in
426 the design, analysis or writing of this article.

427 Logan Manikam is funded by a National Institute for Health Research (NIHR) Doctoral

For peer review only

Research Fellowship (DRF-2014-07-005). Monica Lakhanpaul is funded by the NIHR Collaboration for Leadership in Applied Health Research and Care (CLAHRC) North Thames at Bart's Health NHS Trust.

Authors' contributions

ML, DB, LC, NR, NJ, MM, MJ made substantial contributions to the conception and design of the study. ML was responsible for the overall direction of the project. ML, NR, DB, LC, NH, NJ, MM, CHW, MJ contributed to the analysis and interpretation of data. ML, DB, LC, NR, NJ, MM, MJ contributed to the systematic evidence synthesis. ML, DB, LC, NR, NJ, MM designed and delivered the participatory workshops. ML, DB, LC, NR, NH, NJ, MM, CHW, LM, TH, MJ contributed to the drafting of the report and have given final approval of this report.

Acknowledgements

The authors would like to express their thanks to the following: All families, children, community members and healthcare professionals who participated. The community facilitators and advisory committees.* Professor Jonathan Grigg who contributed to the conception and design of the study and gave advice throughout the project. External advisor, Professor Mike Thomas. Maya Lakhanpaul, Chelsea Huddlestone and Aadil Ali who were our youth facilitators. Ms Gill Perkins and Jo Wilson who assisted with the reviewing of the literature for the systematic evidence synthesis in phase 1. Mrs Stephanie Langan, Mrs Elaine Huddlestone and Mrs Liz Knight. Mrs Emma L. Angell for coding and summarising interview data. Ms Emma Alexander for her thoughtful comments on the draft manuscript. University of Leicester, Leicester Royal Infirmary and the Children's Assessment Unit, Leicester Community Child Health Services, all participating community centres, Mosques

For peer review only

451 and Gurdwaras, organisations, creches*, LCEHR. Asthma UK, with particular thanks to Brigid Hall and Leanne Metcalf. The PCRN and CLRN and the Leicester Community Children's Partnership. Dr Jo Forster. Dr Neena Lakhani. Dr Marian Carey. De Montfort University. Staff at our recruitment sites.*

455 This research was supported by the National Institute for Health Research (NIHR) Collaboration for Leadership in Applied Health Research and Care North Thames at Bart's Health NHS Trust (NIHR CLAHRC North Thames). The views expressed in this article are those of the author(s) and not necessarily those of the NHS, the NIHR, or the Department of Health and Social Care.

461 *Refer to published report for further details

For peer review only - http://bmjopen.bmj.com/site/about/guidelines.xhtml

References:

1. Asthma UK. Asthma facts and statistics. athttps://www.asthma.org.uk/about/media/facts-and-statistics; Date accessed: 21/08/2018
2. Netuveli, G. *et al.* Ethnic variations in UK asthma frequency, morbidity, and health-service use: a systematic review and meta-analysis. *Lancet* **365,** 312–317 (2005).
3. Asthma UK. The asthma divide : inequalities in emergency care for people with asthma in England. - Abstract - Europe PMC. *2012*
4. Kuehni, C. E., Strippoli, M.-P. F., Low, N., Brooke, A. M. & Silverman, M. Wheeze and asthma prevalence and related health-service use in white and south Asian pre-schoolchildren in the United Kingdom. *Clin. Exp. Allergy* **37,** 1738–1746 (2007).
5. Ahmed, S., Steed, L., Harris, K., Taylor, S. J. C. & Pinnock, H. Interventions to enhance the adoption of asthma self-management behaviour in the South Asian and African American population: a systematic review. *npj Prim. Care Respir. Med.* **28,** 5 (2018).
6. Lakhanpaul, M. *et al.* A systematic review of explanatory factors of barriers and facilitators to improving asthma management in South Asian children. *BMC Public Health* **14,** 403 (2014).
7. Bailey, E., Kruske, S., Morris, P., Cates, C. & Chang, A. Culture-specific programs for children and adults from minority groups who have asthma. *Cochrane Database Syst. Rev.* CD006580 (2008). doi:10.1002/14651858.CD006580.pub2
8. Hussain-Gambles, M. *et al.* Involving South Asian patients in clinical trials. *Health Technol. Assess.* **8,** iii, 1-109 (2004).
9. UNICEF. *Convention on the Rights of the Child*. (2010).
10. Lakhanpaul, M. *et al.* The use of a collaborative structured methodology for the development of a multifaceted intervention programme for the management of asthma (the MIA project), tailored to the needs of children and families of South Asian origin: a community-based, participatory study. *Heal. Serv. Deliv. Res.* **2,** 1–226 (2014).
11. Griffith, R. What is Gillick competence? *Hum. Vaccin. Immunother.* **12,** 244–7 (2016).
12. Lakhanpaul, M. *et al.* A qualitative study to identify parents' perceptions of and barriers to asthma management in children from South Asian and White British families. *BMC Pulm. Med.* **17,** 126 (2017).
13. Guevara, J. P., Wolf, F. M., Grum, C. M. & Clark, N. M. Effects of educational interventions for self management of asthma in children and adolescents: systematic review and meta-analysis. *BMJ* **326,** 1308–9 (2003).
14. Palmer, L. J. *et al.* Independent Inheritance of Serum Immunoglobulin E Concentrations and Airway Responsiveness. *Am. J. Respir. Crit. Care Med.* **161,** 1836–1843 (2000).
15. Duffy, D. L., Martin, N. G., Battistutta, D., Hopper, J. L. & Mathews, J. D. Genetics of Asthma and Hay Fever in Australian Twins. *Am. Rev. Respir. Dis.* **142,** 1351–1358 (1990).
16. British Thoracic Society & Scottish Intercollegiate Guidelines Network. *British guideline on the management of asthma*. (2016).
17. Jones, C. O., Qureshi, S., Rona, R. J. & Chinn, S. Exercise-induced bronchoconstriction by ethnicity and presence of asthma in British nine year olds. *Thorax* **51,** 1134–6 (1996).
18. Cohen, R., Franco, K., Motlow, F., Reznik, M. & Ozuah, P. O. Perceptions and attitudes of adolescents with asthma. *J. Asthma* **40,** 207–11 (2003).

For peer review only

For peer review only - http://bmjopen.bmj.com/site/about/guidelines.xhtml

19. Callery, P., Milnes, L., Verduyn, C. & Couriel, J. Qualitative study of young people's and parents' beliefs about childhood asthma. *Br. J. Gen. Pract.* **53,** 185–90 (2003).

20. Macrae, H. Managing courtesy stigma: the case of Alzheimer's disease. *Sociol. Health Illn.* **21,** 141–9889 (1999).

21. Punch, S. Research with Children: The Same or Different from Research with Adults? *Childhood* **9,** 321–341 (2002).

22. Gardner, H. & Randall, D. The effects of the presence or absence of parents on interviews with children. *Nurse Res.* **19,** 6–10 (2012).

For peer review only

Table 1: Demographic information of semi-structured interview participants

	South Asian children (%)	White British children (%)
Total number	33	14
Male	20 (61)	8 (57)
Female	13 (39)	6 (43)
BTS grading		
Level 1	7 (21)	3 (21)
Level 2	17 (52)	8 (57)
Level 3	6 (18)	3 (21)
Level 4	3 (9)	0
Ethno-religious/ethnic group		
Indian Gujarati Hindu	6 (18)	-
Indian Gujarati Muslim	4 (12)	-
Indian Gujarati Jain	1 (3)	-
Indian Punjabi Sikh	6 (18)	-
Indian Punjabi Hindu	1 (3)	-

For peer review only

For peer review only - http://bmjopen.bmj.com/site/about/guidelines.xhtml

**Bangladeshi Muslim**	**9 (27)**	**-**
**Pakistani Muslim**	**6 (18)**	**-**
**White British**	**-**	**13 (93)**
**White British (English)**		**1 (7)**
**White British (Wales)**	**-**	**0**
**White British (Scottish)**	**-**	**0**
**White British (Northern Irish)**	**-**	**0**
**Religion**		
**Hindu**	**7 (21)**	**0**
**Muslim**	**19 (58)**	**0**
**Sikh**	**6 (18)**	**0**
**Jain**	**1 (3)**	**0**
**Christian**	**0**	**6 (43)**
**Atheist**	**0**	**1 (7)**
**No religious belief**	**0**	**4 (29)**
**Not specified**	**0**	**3 (21)**

For peer review only

**Age**		
**5 years old**	**2 (6)**	**2 (14)**
**6 years old**	**5 (15)**	**2 (14)**
**7 years old**	**5 (15)**	**0**
**8 years old**	**4 (12)**	**4 (29)**
**9 years old**	**3 (9)**	**1 (7)**
**10 years old**	**5 (15)**	**4 (29)**
**11 years old**	**5 (15)**	**1 (7)**
**12 years old**	**3 (9)**	**0**
**Not specified**	**1 (3)**	**0**
**Parental/ carer highest level of education achieved ***		
**Primary school**	**4 (8)**	**0**
**Secondary school**	**21 (43)**	**11 (37)**
**College education**	**3 (6)**	**3 (10)**
**University**	**20 (41)**	**14 (47)**
**Not specified**	**1 (2)**	**2 (7)**

For peer review only - http://bmjopen.bmj.com/site/about/guidelines.xhtml

*** Numbers may not equal to the total number of children interviewed as either one or both parents responded to the questionnaire**

For peer review only

Supplementary file 1: Semi-structured interview question schedule

Question schedule for children

Hello. Thank you very much for agreeing to speak to me. So I'm (interviewer), and this is an interview for the MIA research study. We' re going to talk about your breathing. I'm going to ask you things like what you remember from when you were very little, and I'm going to ask you if you remember what it's like now. I'll also ask some questions about what happens when you have a bad day, medicines you take and what other people say about your asthma. If you don't know the answer, you can just guess. There's no right or wrong answer.

1. When did your breathing problems first start?
    - Initial symptoms/recognition
    - Who/how diagnosed
    - Information/support provided
    - Barriers to diagnosis
        - Seeking help
        - HCP attitude
        - Access to service
2. People have lots of different Ideas about why some children get wheezy. Why do you think experience wheeziness?
    - Triggers vs. cause
    - Heritability
    - Contagion
    - Gods will
    - Diet

For peer review only

3. What do you think causes asthma/ wheezing?
4. Could you describe what's your asthma like on a day-to-day basis
    - Symptoms
    - Impact on day-to-day life
    - Lifestyle restriction
    - Time off work
    - Family life
    - Schooling
5. Who makes the decision about day-to-day things?
    - Mum/dad
    - Child
    - Extended family
6. Could you describe a recent asthma attack? (substitute bad attack if little response)
    - Describe management
        - Use of health services
        - Use of tests/diagnostics
        - Role of extended family in management
7. Problems faces
    - Recognition of need to seek help
    - Access to service A&E/ walk in/ GP
    - Practicalities- carrying medicines, time of day, weekends etc
    - HCP communication/attitudes/ethnicity/ gender
8. What worked well?
    - Good service

For peer review only

- Good person

9. Information provided
    - What
    - From who
    - Useful/not
    - What else wanted
    - Timing
10. Solutions used/wanted
11. Let's talk about treatments. What do you hope treatments will do for your asthma/wheezing?
    - Personal goals of treatment
    - Management vs. cure
    - Concept of asthma control
    - What is good control
12. Do you take any medicines for your asthma/wheezing? (substitute inhalers if no response) (go through medicine one by one)
    - When do you use this/how do you feel about it/any problems getting taking it/ how do you remember to take it/ does everyone in the family know about it/ use in public?
    - For preventer medicines: when do you stop/ who makes decisions/ side effects?
    - Specific attitudes
        - Long term med use
        - Inhaler vs. tablets
        - Steroids

For peer review only

13. Who has helped you learn about wheezing/asthma?
    - Sources of knowledge
    - Skills
    - Overseas advice
    - Role of schools/community centres/religious centres
    - Use of written management plans
14. What do the rest of the family think about your asthma?
    - Who knows/ gets involved
    - What do you think
    - Does anyone offer advice/ do you follow it?
    - Impact on management
15. What about your friends?
    - Do you tell others/ stigma?
    - Would you tell about another illness/specific to asthma?
    - Common beliefs about cause
    - Common attitudes towards asthma/ children with asthma
    - Advice offered/ follow it
    - Has anyone upset you?
    - Do cultural attitudes influence management?
16. Where do you go for help about your asthma?
    - Useful source of care- GP/A&E/walk-in/family and friends/internet/religious leaders
        - Who do you go to?
        - What is the key issue- opening hours, location, relationship, gender, ethnicity

For peer review only

- Problems faced getting help
  - Communication with HCPs
  - Getting prescriptions

17. What would overcome these?

18. Is there anything that you've found particularly helpful in taking care of your asthma?

- What/why?
- Alternative therapies used?
  - What/why
  - Diet
  - Heating/furnishing changes
  - Barriers or facilitator to using different options

19. Is there anything else we could do to help you cope with your asthma?

- What/why/how
  - Specific to your family
  - General suggestions for families and children

For peer review only

Consolidated criteria for reporting qualitative studies (COREQ): 32-item checklist[1]

No	Item	Guide questions/description	Checklist
**Domain 1: Research team and reflexivity**			
**Personal Characteristics**			
**1.**	Interviewer/facilitator	Which author/s conducted the interview or focus group?	Dr Deborah Bird<br>Dr Melanie McFeeters
**2.**	Credentials	What were the researcher's credentials? *E.g. PhD, MD*	See 'authors and institution' Page 1-3, Line 4-72
**3.**	Occupation	What was their occupation at the time of the study?	See 'authors and institution' Page 1-3, Line 4-72
**4.**	Gender	Was the researcher male or female?	Female<br>Page 10, Line 195
**5.**	Experience and training	What experience or training did the researcher have?	Page 10, Line 195-196
**Relationship with participants**			
**6.**	Relationship established	Was a relationship established prior to study commencement?	Yes; via encouraging children to draw and write about their asthma.<br>Page 10, Line 193-194
**7.**	Participant knowledge of the interviewer	What did the participants know about the researcher? e.g. *personal goals, reasons for doing the research*	Supplementary file 1
**8.**	Interviewer characteristics	What characteristics were reported about the interviewer/facilitator? e.g. *Bias, assumptions, reasons and interests in the*	Page 10, Line 194-196. Interviewer characteristics reported are:

		research topic	gender, profession, level of training in research and conflict of interests
**Domain 2: study design**			
**Theoretical framework**			
**9.**	Methodological orientation and Theory	What methodological orientation was stated to underpin the study? *e.g. grounded theory, discourse analysis, ethnography, phenomenology, content analysis*	Semi-structured interview (grounded theory) Page 9, Line 167
**Participant selection**			
**10.**	Sampling	How were participants selected? *e.g. purposive, convenience, consecutive, snowball*	Purposive sampling Page 9, Line 177-179
**11.**	Method of approach	How were participants approached? *e.g. face-to-face, telephone, mail, email*	Face-to-face and telephone Page 9, Line 170-173
**12.**	Sample size	How many participants were in the study?	Page 9, Line 181
**13.**	Non-participation	How many people refused to participate or dropped out? Reasons?	Due to loss of interest, some children did not wish to participate in the interviews (n=2)
**Setting**			
**14.**	Setting of data collection	Where was the data collected? *e.g. home, clinic, workplace*	Family home. Page 10, Line 197
**15.**	Presence of non-participants	Was anyone else present besides the participants and researchers?	Some independently and some with parents. Page 10, Line 197
**16.**	Description of sample	What are the important	Table 1. Page

		characteristics of the sample? *e.g. demographic data, date*	27-30, Line 565-568
	**Data collection**		
**17.**	Interview guide	Were questions, prompts, guides provided by the authors? Was it pilot tested?	Supplementary file 1
**18.**	Repeat interviews	Were repeat interviews carried out? If yes, how many?	No repeat interviews were conducted. Page 10, Line 198-199
**19.**	Audio/visual recording	Did the research use audio or visual recording to collect the data?	Interviews were digitally voice-recorded. Page 10, Line 197-198
**20.**	Field notes	Were field notes made during and/or after the interview or focus group?	Fields notes were taken. Page 10, Line 198
**21.**	Duration	What was the duration of the interviews or focus group?	Interviews typically lasted between 20-60 minutes [1]
**22.**	Data saturation	Was data saturation discussed?	No
**23.**	Transcripts returned	Were transcripts returned to participants for comment and/or correction?	Transcripts were not returned to children for comments/ corrections. Page 10, Line 202-203
	**Domain 3: analysis and findings**		
**Data analysis**			

[1] Monica Lakhanpaul and others, *The Use of a Collaborative Structured Methodology for the Development of a Multifaceted Intervention Programme for the Management of Asthma (the MIA Project), Tailored to the Needs of Children and Families of South Asian Origin: A Community-Based, Partici* (NIHR Journals Library, 2014) <http://dx.doi.org/10.3310/HSDR02280>.

**24.**	Number of data coders	How many data coders coded the data?	There were two data coders. Page 10, Line 205
**25.**	Description of the coding tree	Did authors provide a description of the coding tree?	Page 10-11, Line 204-216
**26.**	Derivation of themes	Were themes identified in advance or derived from the data?	Page 10-11, Open coding. Line 204-216
**27.**	Software	What software, if applicable, was used to manage the data?	NVivo (QSR International, Warrington, UK). Page 10 Line 202
**28.**	Participant checking	Did participants provide feedback on the findings?	Transcripts were not returned to children for comments/ corrections. Page 10, Line 202-203
**Reporting**			
**29.**	Quotations presented	Were participant quotations presented to illustrate the themes / findings? Was each quotation identified? e.g. *participant number*	*Results* Page 12-17, Line 225-358
**30.**	Data and findings consistent	Was there consistency between the data presented and the findings?	Discussion. Page 18-20, Line 365-420
**31.**	Clarity of major themes	Were major themes clearly presented in the findings?	Discussion. Page 18-20, Line 365-420
**32.**	Clarity of minor themes	Is there a description of diverse cases or discussion of minor themes?	Discussion. Page 18-20, Line 365-420

1. Allison Tong, Peter Sainsbury, Jonathan Craig; Consolidated criteria for reporting qualitative research (COREQ): a 32-item checklist for interviews and focus groups, *International Journal for Quality in Health Care*, Volume 19, Issue 6, 1 December 2007, Pages 349–357, https://doi.org/10.1093/intqhc/mzm042

For peer review only

A qualitative study to identify ethnic-specific perceptions of and barriers to asthma management in South Asian and White British children with asthma

Journal:	*BMJ Open*
Manuscript ID	bmjopen-2018-024545.R2
Article Type:	Research
Date Submitted by the Author:	23-Nov-2018
Complete List of Authors:	Lakhanpaul, Monica; University College London Institute of Child Health, Population, Policy and Practice ; Whittington Health NHS Trust, Department of Paediatrics<br>Culley, Lorraine; De Montfort University, School of Applied Social Sciences<br>Huq, Tausif; King's College London, School of Medical Education<br>Bird, Deborah ; London North West Healthcare NHS Trust, Ealing Child Development Team<br>Hudson, Nicky; DeMontfort University, School of Applied Social Science<br>Robertson, Noelle ; University of Leicester Centre for Medical Humanities, Clinical Psychology<br>McFeeters, Melaine ; NHS England , Specialised Commissioning East Midlands<br>Manikam, Logan; University College London Institute of Epidemiology and Health Care, Department of Epidemiology & Public Health<br>Johal , Narynder ; Parent representative<br>Hamlyn-Williams , Charlotte ; University College London Institute of Child Health, Population, Policy and Practice<br>Johnson, Mark; DeMonfort University, Mary Seacole Research Centre
**Primary Subject Heading**:	Paediatrics
Secondary Subject Heading:	Respiratory medicine, Public health, Health services research, Qualitative research
Keywords:	Asthma < THORACIC MEDICINE, Community child health < PAEDIATRICS, QUALITATIVE RESEARCH, PAEDIATRICS, PUBLIC HEALTH

SCHOLARONE™
Manuscripts

A qualitative study to identify ethnic-specific perceptions of and barriers to asthma management in South Asian and White British children with asthma

Corresponding author

1. **Professor Monica Lakhanpaul**

Institutional address: Population, Policy and Practice, UCL Great Ormond Street Institute of Child Health, 30 Guildford Street, London WC1N 1EH, UK

Email address: m.lakhanpaul@ucl.ac.uk

Telephone number: +44 (0)20 7905 2259

Professor of Integrated Community Child Health

Consultant Paediatrician

Whittington Health NHS Trust

2. Professor Lorraine Culley

Institutional address: School of Applied Social Sciences, De Montfort University, The Gateway, Leicester, LE1 9BH, UK

Email address: lac@dmu.ac.uk

Telephone number: +44 (0)116 257 7753

3. Tausif Huq

Institutional address: King's College London, Guy's King's and St Thomas' Hospital (GKT) School of Medicine, New Hunt's House, Guy's Campus, London, SE1 1UL, UK

For peer review only

Email address: tausif.huq@kcl.ac.uk

Telephone number: +44 (0)7454003467

4. Dr Deborah Bird

Institutional address: Ealing Child Development Team, Carmelita House, 21-22 The Mall, Ealing, W5 2PL

Email address: deborah.bird@nhs.net

Telephone number: +44 (0) 208 825 8700

5. Prof. Nicky Hudson

Institutional address: School of Applied Social Sciences. De Montfort, University, The Gateway, Leicester, LE1 9BH, UK

Email address: nhudson@dmu.ac.uk

Telephone number: +44 (0)116 207 8766

6. Prof. Noelle Robertson

Institutional address: Clinical Psychology, University of Leicester, Centre for Medicine, Lancaster Road, Leicester, LE1 7HA, UK

Email address: nr6@le.ac.uk

Telephone number: +44 (0)116 223 1617/1648

7. Dr Melanie McFeeters

Institutional address: Specialised Commissioning East Midlands, NHS England, Fosse House, 6 Smith Way, Grove Park, Enderby, Leicestershire, LE19 1SX, UK

For peer review only

Email address: Melanie.Mcfeeters@nhs.net

Telephone number: +44 (0) 7717 720185

8. Dr Logan Manikam

Institutional address: Department of Epidemiology & Public Health, UCL Institute of Epidemiology & Healthcare, 1-19 Torrington Place, London WC1E 7HB, UK

Email address: logan.manikam.10@ucl.ac.uk

Telephone number: +44 (0)7868721152

9. Narynder Johal

Institutional address: Parent representative, Leicester, UK

Email address: narynder@hotmail.com

Telephone number: n/a

10. Dr Charlotte Hamlyn-Williams

Institutional address: Population, Policy and Practice, UCL Great Ormond Street Institute of Child Health, 30 Guildford Street, London WC1N 1EH, UK

Email address: c.hwilliams@outlook.com

Telephone number: +44 (0)7748282923

11. Professor Mark R D Johnson

Institutional address: Mary Seacole Research Centre, De Montfort University, The Gateway, Leicester, LE1 9BH, UK

Email address: mrdj@dmu.ac.uk

For peer review only

Telephone number: +44 (1) 676 532389

Abstract

Objective: This paper draws on the data from the Management and Interventions for Asthma (MIA) study to explore the perceptions and experiences of asthma in British South Asian children using semi-structured interviews. A comparable cohort of White British children was recruited to identify whether any emerging themes were subject to variation between the two groups so that generic and ethnic-specific themes can be identified for future tailored intervention programmes for South Asian children with asthma.

Setting: South Asian and White British children with asthma took part in semi-structured interviews in Leicester, UK.

Participants: 33 South Asian and 14 White British children with asthma aged 5-12 years were interviewed.

Results: Both similar and contrasting themes emerged from the semi-structured interviews. Interviews revealed considerable similarities in the experience of asthma between the South Asian and White British children, including the lack of understanding of asthma (often confusing trigger with cause), lack of holistic discussions with healthcare professionals (HCPs), an overall neutral or positive experience of interactions with HCPs, the role of family in children's self-management and the positive role of school and friends. Issues pertinent to South Asian children related to the higher likelihood of feeling embarrassed and attributing physical activity as a trigger for asthma symptoms.

For peer review only

95 **Conclusions**: The two ethnic-specific factors revealed by the interviews are significant in children's self-management of asthma, and therefore indicate the need for a tailored intervention in South Asian children.

98 **Keywords:** Asthma, Paediatrics, Qualitative Research, Collaborative research Community child health, Public health

100 **Word count:** Abstract- 234 words, Text- 2990 words, Table- 1

101 **Article summary**

102 **Strengths and limitations of this study:**

103 - An unequal power relationship between adult researchers and child participants has been mitigated through the use of 'child-friendly' data collection methods.

106 - The study was not resourced to analyse children's drawings, although if done systematically, this may provide additional useful insights into children's understanding and experience of asthma.

109 - Some children declined to participate in the activities provided, such as drawing, and the activities themselves increased the length of interview time.

111

For peer review only

Introduction

Asthma is one of the most prevalent chronic conditions in childhood. Estimates in the UK suggests 1.1 million, or one in ten children will experience asthma at some point in their childhood[1]. Evidence shows that children from South Asian communities experience under-recognition of asthma symptoms, worse disease severity and increased attendance to emergency department, exceeding the rates of their White British counterparts[2–4]. However, there is no evidence to indicate South Asian children experience more severe asthma or have a genetic predisposition to severe asthma attacks[2–4].

Barriers to effective asthma management in South Asian children are well recognised in the literature[2–4]. However, many health interventions aimed at mitigating these barriers have failed to improve asthma outcomes, often ascribed to a lack of cultural sensitivity and a failure to recognise the ethno-religious heterogeneity within the South Asian community[5]. A systematic review by Lakhanpaul *et al*. identified several cultural and structural barriers to optimal asthma management in South Asian children, including diverse knowledge of asthma, non-acceptance and parental denial of asthma[6]. It highlighted the importance of identifying the nature and mechanism of these barriers so that ethnically-tailored interventions can be developed. 'Tailored' interventions consider individual cultural influences, and are more effective than 'modified' interventions, which modify an intervention designed for a majority population[6,7].

For peer review only

There is also a remarkable under-representation of UK minority ethnic children in all aspects of asthma research, thus specific ethnic-specific and religious-specific factors are rarely integrated into health services[8]. This is despite international (*UN Convention on the Rights of the Child*[9]) and national policy mandates (including the Children Act 1989 and 2004 affecting England and Wales) to increase precedence of the views and opinions of children and young people (CYP). As CYP are not a homogenous group and have varying health needs depending on backgrounds, capabilities and interests, it is important that their views are heard, especially in childhood asthma.

Context of the current study: the MIA project

This paper is drawn from the Management and Intervention for Asthma (MIA) study- carried out in an urban area in Leicester, UK. It had an overall aim to develop an intervention-planning framework for British South Asian children with asthma, guided by a participatory collaborative method which involved individuals and communities defining problems and developing solutions[10].

This paper reports on one of the major phases of the MIA study: to explore the perceptions and experiences of asthma in British South Asian children by semi-structured interviews. A comparable cohort of White British children was recruited to identify whether any emerging themes were subject to variation between the two groups, so that generic and ethnic-specific themes could be identified for future tailored intervention programmes in British South Asian children with asthma. Children's interviews were complemented by interviews with parents/carers, community members and healthcare professionals (HCPs), the results of which are discussed elsewhere[10].

For peer review only - http://bmjopen.bmj.com/site/about/guidelines.xhtml

Methods

This study adopted a qualitative methodology using semi-structured interviews.

Participant sampling

Families with children aged 5-12 years with a diagnosis of asthma were included (Table 1). Participants were purposively sampled via two routes: via healthcare (e.g. general practice) and the community, facilitated by community facilitators (CFs), who were bilingual members of the South Asian community, trained as lay researchers to assist with the study.

Recruitment ensured a proportional representation of six major South Asian ethno-religious groups (Table 1; these terms describe ethno-religious identity and not participants' nationality), with parents asked to assign children's demographic information. From the recruited families, purposive sampling was used to recruit children (matching age, sex and asthma severity according to British Thoracic Society guidelines[10]). In some instances, more than one child was recruited from a family.

Thirty-three South Asian children and 14 White children were interviewed. BTS grading allowed a wide range of asthma experiences to be captured (Table 1).

For peer review only - http://bmjopen.bmj.com/site/about/guidelines.xhtml

Consent and assent

Adults (parents/legal guardians) consented on behalf of their children. Assessment of 'Gillick competency' was used to obtain consent from children aged 10-12-years[11]. Younger children were judged by the research team on their ability to provide assent orally where appropriate.

Data collection

A question schedule was developed from the findings of an initial scoping review and by external advisors (Supplementary file 1)[10]. Children also produced drawings and text during the interviews which guided interview questions. Interviews were carried out by two female researchers (a paediatrician and a social scientist, both trained in qualitative methods and not involved in their care). Interviews took place in children's family homes with some choosing to give interviews with their parents. Interviews were digitally recorded, transcribed and field notes were taken. No repeat interviews were conducted.

Data analysis

Interview transcripts were analysed according to the principles of interpretive thematic analysis using NVivo (QSR International, Warrington, UK)[10]. Transcripts were not returned to children for comments/corrections.

A detailed explanation of the thematic analysis can be found in previous publications[10,12]. Briefly, two analysts independently analysed the transcripts to create a framework of thematic categories from both interviews. The thematic frameworks of South Asian and White British children's data were then closely compared and amended to ensure consistency of meaning across both datasets to

For peer review only

create a comprehensive coding framework. All transcripts were then systematically coded using the resultant framework.

Patient and public involvement

Children were not directly involved in the design, recruitment or conduct of the study but participated in the development of an intervention framework following the interviews, in a series of community-based workshops[10]. Parents were included as members of the research team (NJ, as co-applicant) and as members of the advisory group. Finally, a number of dissemination events were held in the community involving South Asian and White British children.

For peer review only

Results

The key themes arising from the exploration of the interviews were: understandings of asthma; children's self-management of asthma; perception of asthma; management in school; the role of friends; and interactions with healthcare providers.

Children's understandings of asthma

Fourteen South Asian children (42%) and six White British children (43%) explained what they thought to be the cause for asthma, although most were uncertain. Some children attributed causes of asthma to triggers such as the weather, the environment, or viruses.

"Well I think it's where if you are near something that triggers it off your lungs might tighten together a little bit and stop you breathing and lock up your air pipe a little bit so you will get quite wheezy" (White British boy)

"As mum keeps saying over and over again, the damp weather, […] I may have caught asthma from the damp weather…". (Indian girl)

Two South Asian and two White British children thought that asthma had a hereditary component.

"I know it runs in families and stuff because me and my mum have got it". (White British girl)

"Probably because like someone in their family before like your grandma or

For peer review only - http://bmjopen.bmj.com/site/about/guidelines.xhtml

grandfather had it [...]" (Punjabi boy)

Children's interactions with the healthcare system

Seven South Asian (21%) and seven White British children (50%) could not recall the time they were diagnosed with asthma. Furthermore, children were more likely to remember their recent interactions with HCPs rather than those in the past.

Overall, children's interactions with HCPs were described broadly as positive or neutral. Children were more likely to recall their visits to their general practitioner (GP) since most had visited recently. Positive comments focused on their GP's friendly approach and communications, including explaining asthma and medications. Three negative comments related to "horrible tasting tablets", misdiagnosis of hay fever and being "ignored" after initiating nebuliser.

"He [doctor], he'll tell you what's happening, what's wrong, and if you have asthma or if you don't have asthma, and he'll tell you if you need a, the stuff for the inhaler you when you put it in." (Pakistani boy)

In both groups, experiences and perceptions of secondary care were generally less positive, often involving negative emotional descriptions and related to being treated for acute asthma attacks. Nevertheless, the quality of interactions with individual HCPs remained positive.

"Just a little bit scary. […] Because sometimes you have to have injections. [The nurses are] so kind. […] So what [the doctors] do injections they, when they've done it, [the nurses] cheer me up when I cry." (Indian girl)

South Asian and White British children did not recall having a holistic discussion with

For peer review only - http://bmjopen.bmj.com/site/about/guidelines.xhtml

the HCPs such as discussions about school, physical activity and quality of life. Some also recalled GPs not involving children during consultations.

"[Doctors should talk to] both [me and my parents][…] Like, to explain […] what is it happening, […] after explaining to the parents[…]" (Bangladeshi girl)

Children's self-management of asthma

Twenty-nine South Asian (89%) and four White British children (29%) spoke about self-assessing symptoms and self-management, especially as they became older. In both groups, parents were described as being responsible for reminding and instructing children to take inhalers and facilitating the process of self-management.

The families' response to an asthma attack appeared to mediate a child's symptom perception. Both groups of children reported that parents, especially their mothers, played a vital role in managing acute attacks by spotting symptoms and encouraging inhaler use.

"My mum tells me...how many puffs...when she wants to do something else, […]she...she...she says how many times to do it. And then I start doing it. […]Sometimes my dad helps me when I'm like stuck. (Indian girl)

It was evident that the older children devised their own system of self-management, with the help of their parents.

"…so always have two inhalers of the same colour, like… if this one ran out then I'd tell my Mum or Dad or sister…" (Indian boy)

Overall, parents (especially mothers) in both groups of children were felt to play an

For peer review only

important role in mediating children's symptom perception and self-management of routine and acute attacks, with no specific ethnic differences.

Children's perception of the impact of asthma

One of the biggest impacts of asthma on children as reported was how it affected their ability to participate in sporting activities and physical education classes at school. South Asian children more frequently attributed physical activity as a trigger for an asthma attack (n=29, 89%) than White British children (n=7, 50%). Common symptoms included breathlessness and coughing, resulting in children either sitting out or avoiding activities altogether.

> *"Because you know when I run the wheeziness starts and make me cough […] So that' s why I take the inhaler sometimes when we come back from sports or anything." (Indian boy)*

> *"I have had to do it once [take inhaler] before but it was sports day […]."* (*White British girl*)

The most commonly reported emotions were embarrassment (n=11 South Asian; n=3 White British), fear (n=3 South Asian; n=2 White British), upset (n=7 South Asian; n=1 White British) and unfairness (n=2 South Asian; n=2 White British). South Asian children were more likely to report embarrassment in relation to sports, school and taking medication than White British children. It was described however with some emotional distance as an embarrassing condition rather than children being embarrassed (See *School and friends*).

"[…] she's grown up, she says, It's embarrassing to carry it [inhaler], mum. " (Mother of a Bangladeshi girl)

For peer review only - http://bmjopen.bmj.com/site/about/guidelines.xhtml

"That I have to cough when my sister doesn't cough, or I have to have more medication than she has to have. It's a bit annoying sometimes but you get used to it." (White British girl)

Both groups of children expressed few positive emotions and when asked if they had one wish, many reported that they wanted asthma to 'go away'. A few children did find some positives in their experience of asthma management.

"The good thing is erm […] I think I've improved in my confidence and my determination […]." (Bangladeshi boy)

School and friends

Peers and friends were described as having an important role in children's experience and management of asthma. Having friends who understood their asthma, and in some cases, could help if needed, was deemed important for both South Asian and White British children.

"At school it doesn't affect me much, people don't tease me or anything… , their friends are aware of my asthma, some of them have asthma themselves and they help me". (Pakistani boy)

"… they [friends] mostly help me, like they usually go get it [inhaler] for me." (White British girl)

Asthma was considered a reason to feel different or 'not normal', thus a small number of children described strategies to manage the amount of information they disclosed to their peers.

For peer review only

"I have to explain it a lot . . . they understand I have got it and I think they are alright because they know it's not catching." (White British girl)

One White British girl felt that using her inhaler at school attracted unwanted attention and one South Asian child reported being teased about his asthma.

Suggestions for service improvements

Some children discussed ways in which knowledge of asthma could be improved and offered ideas for future interventions. Children reported wanting to speak to someone who had experienced asthma, either living with asthma or a HCP. Children also described ways in which information can be delivered.

"Pictures maybe." (Indian boy)

"A presentation[. . .]. They tell you things that I did not know like in more detail.[. . .] The chemist and the GP were ok but the school one explained it best and easy to understand." (Indian boy)

"They [school] could do a little show about asthma or something like that […]" (White British girl)

For peer review only

Discussion

Semi-structured interviews with children in this study provided an opportunity for those affected by asthma to offer their experiences and perceptions of asthma management. The interviews revealed that some perceived challenges facing children with asthma may be common regardless of ethnicity. However, certain experiences appeared to be more relevant to South Asian children, in particular feelings of embarrassment and attributing physical activity as a trigger for asthma symptoms.

Children as young as 6-years-old are able to self-manage asthma with parents having a positive intermediary effect[13]. Therefore, knowledge of asthma will support and nurture the practice of self-management. However, both groups of children expressed confusions and misconceptions about the causes, triggers, and nature of asthma[14,15]. These findings indicate that the HCPs may not be adequately delivering health information and/or checking the understanding of those receiving the information. Consequently, the need for an improved information provision is echoed by the children in the suggestions they offered for improved asthma support.

Interactions with HCPs were mostly reported as positive or neutral by the South Asian and White British children and did not reveal any ethnic-specific barriers to communication. Some children described being ‘left out’ during doctors’ consultations, which were often directed at their parents. Children often recalled providing a clinical history and being examined but did not recollect having any holistic discussions. This is consistent with the data collected from an earlier phase of the MIA study[10] which found that HCPs tended to focus on addressing

For peer review only - http://bmjopen.bmj.com/site/about/guidelines.xhtml

medications with limited discussions about holistic management. However, the lack of recall of interactions with HCPs may also relate to children's age and the time of their recent interaction.

Physical activity is recommended within asthma management guidelines[16]. Our results show that the South Asian children are more likely to attribute physical activity as a trigger for asthma symptoms than the White British children. Indeed, it has been reported that South Asian children are 3.6 times more likely to suffer from exercise-induced bronchoconstriction than White British children[17]. Regardless of the underlying mechanism, our results may indicate the need for a tailored approach to change the perceptions of physical activity in South Asian children.

A desire to be normal was commonly expressed by the children, echoing previous studies examining the psychosocial aspects of asthma[18,19]. The children struggled to talk positively about asthma, with negative emotions equally apparent in both groups. However, the self-conscious emotion of embarrassment was more likely to be experienced by the South Asian children, and often related to day-to-day activities such as sports, or taking medication when the children were seen as visibly different, rather than having asthma per se. However, this must be interpreted cautiously as there were more 'older' children (11-12-year olds) in the South Asian cohort compared to White British children, and this perception may be a reflection of the age difference. Nevertheless, the sense of embarrassment may be compounded by an indirect experience of asthma-related stigmatisation ("courtesy stigma"[20]) from the wider South Asian community[10,12]. Children who feel embarrassed are less likely to carry or use inhalers with an overall negative impact on self-management[18]. Therefore, a tailored intervention to tackle emotional affects in South Asian children

For peer review only - http://bmjopen.bmj.com/site/about/guidelines.xhtml

should be considered.

Challenges and limitations

Semi-structured interviews with children can involve unequal power dynamics between an adult researcher and child participant with differing power dynamics at play when interviewed with or without the presence of parents[21,22]. Efforts were made to minimize this impact by using child-friendly strategies[10]. The children's age range (5-12 years) is also a limiting factor (Table 1), potentially influencing asthma experience and recall. Furthermore, interviewing young children also increases costs, both in terms of resources such as child-friendly interview/involvement resources, and due to increased time needed to establish trust and maintain interest.

Policy Practice and Conclusion

Interviews with children from South Asian and White British communities revealed considerable similarities in their experiences and perceptions of asthma and its management. It is therefore important to work with both South Asian and White British children to design interventions which take into consideration the important common issues raised in this qualitative study. However, two ethnic-specific themes emerged from the interviews, which suggest that there may also be a need for an intervention to include a more tailored component for South Asian children, particularly around physical activity and social stigma. The MIA study also included interviews and focus groups with parents/carers, HCPs and wider community members, revealing further ethnic-specific barriers and facilitators to asthma management in South Asian children which can indirectly affect children's experience[10]. Overall, these data can contribute to the development of a multifaceted

For peer review only

tailored intervention to improve the management of asthma in South Asian children.

For peer review only

For peer review only - http://bmjopen.bmj.com/site/about/guidelines.xhtml

Abbreviations

HCPs: Healthcare Professionals; GPs: General Practitioners; PE: Physical education

Declarations

Data sharing statement

No additional data are available

Ethics approval and consent to participate

Ethical approval was gained through the local NHS ethics committee (ref. 09/H0401/85), University ethics and NHS R&D approval. All participants gave written or verbal consent to participate in the study.

Competing interests

During the MIA project, Monica Lakhanpaul was appointed as a National Institute for Health and Care Excellence (NICE) Fellow, member of the NHS Evidence advisory board, the Health Technology Assessment advisory panel and the Drugs and Therapeutics Bulletin editorial panel.

Funding

This project was funded by the National Institute for Health Research. Health Services and Delivery Research (HS&DR) programme (project number 09/2001/19). NIHR had no role in the design, analysis or writing of this article.

Logan Manikam is funded by a National Institute for Health Research (NIHR) Doctoral

For peer review only

Research Fellowship (DRF-2014-07-005). Monica Lakhanpaul is funded by the NIHR Collaboration for Leadership in Applied Health Research and Care (CLAHRC) North Thames at Bart's Health NHS Trust.

Authors' contributions

ML, DB, LC, NR, NJ, MM, MJ made substantial contributions to the conception and design of the study. ML was responsible for the overall direction of the project. ML, NR, DB, LC, NH, NJ, MM, CHW, MJ contributed to the analysis and interpretation of data. ML, DB, LC, NR, NJ, MM, MJ contributed to the systematic evidence synthesis. ML, DB, LC, NR, NJ, MM designed and delivered the participatory workshops. ML, DB, LC, NR, NH, NJ, MM, CHW, LM, TH, MJ contributed to the drafting of the report and have given final approval of this report.

Acknowledgements

The authors would like to express their thanks to the following: All families, children, community members and healthcare professionals who participated. The community facilitators and advisory committees.* Professor Jonathan Grigg who contributed to the conception and design of the study and gave advice throughout the project. External advisor, Professor Mike Thomas. Maya Lakhanpaul, Chelsea Huddlestone and Aadil Ali who were our youth facilitators. Ms Gill Perkins and Jo Wilson who assisted with the reviewing of the literature for the systematic evidence synthesis in phase 1. Mrs Stephanie Langan, Mrs Elaine Huddlestone and Mrs Liz Knight. Mrs Emma L. Angell for coding and summarising interview data. Ms Emma Alexander for her thoughtful comments on the draft manuscript. University of Leicester, Leicester Royal Infirmary and the Children's Assessment Unit, Leicester Community Child Health Services, all participating community centres, Mosques

For peer review only

450 and Gurdwaras, organisations, creches*, LCEHR. Asthma UK, with particular thanks to
451 Brigid Hall and Leanne Metcalf. The PCRN and CLRN and the Leicester Community
452 Children's Partnership. Dr Jo Forster. Dr Neena Lakhani. Dr Marian Carey. De Montfort
453 University. Staff at our recruitment sites.*

454 This research was supported by the National Institute for Health Research (NIHR)
455 Collaboration for Leadership in Applied Health Research and Care North Thames at Bart's
456 Health NHS Trust (NIHR CLAHRC North Thames). The views expressed in this article are
457 those of the author(s) and not necessarily those of the NHS, the NIHR, or the Department of
458 Health and Social Care.

459

460 *Refer to published report for further details

For peer review only - http://bmjopen.bmj.com/site/about/guidelines.xhtml

References:

1. Asthma UK. Asthma facts and statistics. athttps://www.asthma.org.uk/about/media/facts-and-statistics; Date accessed: 12/11/2018
2. Netuveli, G. *et al.* Ethnic variations in UK asthma frequency, morbidity, and health-service use: a systematic review and meta-analysis. *Lancet* **365,** 312–317 (2005).
3. Asthma UK. The asthma divide : inequalities in emergency care for people with asthma in England. - Abstract - Europe PMC. *2012*
4. Kuehni, C. E., Strippoli, M.-P. F., Low, N., Brooke, A. M. & Silverman, M. Wheeze and asthma prevalence and related health-service use in white and south Asian pre-schoolchildren in the United Kingdom. *Clin. Exp. Allergy* **37,** 1738–1746 (2007).
5. Ahmed, S., Steed, L., Harris, K., Taylor, S. J. C. & Pinnock, H. Interventions to enhance the adoption of asthma self-management behaviour in the South Asian and African American population: a systematic review. *npj Prim. Care Respir. Med.* **28,** 5 (2018).
6. Lakhanpaul, M. *et al.* A systematic review of explanatory factors of barriers and facilitators to improving asthma management in South Asian children. *BMC Public Health* **14,** 403 (2014).
7. Bailey, E., Kruske, S., Morris, P., Cates, C. & Chang, A. Culture-specific programs for children and adults from minority groups who have asthma. *Cochrane Database Syst. Rev.* CD006580 (2008). doi:10.1002/14651858.CD006580.pub2
8. Hussain-Gambles, M. *et al.* Involving South Asian patients in clinical trials. *Health Technol. Assess.* **8,** iii, 1-109 (2004).
9. UNICEF. *Convention on the Rights of the Child*. (2010).
10. Lakhanpaul, M. *et al.* The use of a collaborative structured methodology for the development of a multifaceted intervention programme for the management of asthma (the MIA project), tailored to the needs of children and families of South Asian origin: a community-based, participatory study. *Heal. Serv. Deliv. Res.* **2,** 1–226 (2014).
11. Griffith, R. What is Gillick competence? *Hum. Vaccin. Immunother.* **12,** 244–7 (2016).
12. Lakhanpaul, M. *et al.* A qualitative study to identify parents' perceptions of and barriers to asthma management in children from South Asian and White British families. *BMC Pulm. Med.* **17,** 126 (2017).
13. Guevara, J. P., Wolf, F. M., Grum, C. M. & Clark, N. M. Effects of educational interventions for self management of asthma in children and adolescents: systematic review and meta-analysis. *BMJ* **326,** 1308–9 (2003).
14. Palmer, L. J. *et al.* Independent Inheritance of Serum Immunoglobulin E Concentrations and Airway Responsiveness. *Am. J. Respir. Crit. Care Med.* **161,** 1836–1843 (2000).
15. Duffy, D. L., Martin, N. G., Battistutta, D., Hopper, J. L. & Mathews, J. D. Genetics of Asthma and Hay Fever in Australian Twins. *Am. Rev. Respir. Dis.* **142,** 1351–1358 (1990).
16. British Thoracic Society & Scottish Intercollegiate Guidelines Network. *British guideline on the management of asthma*. (2016).
17. Jones, C. O., Qureshi, S., Rona, R. J. & Chinn, S. Exercise-induced bronchoconstriction by ethnicity and presence of asthma in British nine year olds. *Thorax* **51,** 1134–6 (1996).
18. Cohen, R., Franco, K., Motlow, F., Reznik, M. & Ozuah, P. O. Perceptions and attitudes of adolescents with asthma. *J. Asthma* **40,** 207–11 (2003).

For peer review only

For peer review only - http://bmjopen.bmj.com/site/about/guidelines.xhtml

19. Callery, P., Milnes, L., Verduyn, C. & Couriel, J. Qualitative study of young people's and parents' beliefs about childhood asthma. *Br. J. Gen. Pract.* **53,** 185–90 (2003).
20. Macrae, H. Managing courtesy stigma: the case of Alzheimer's disease. *Sociol. Health Illn.* **21,** 141–9889 (1999).
21. Punch, S. Research with Children: The Same or Different from Research with Adults? *Childhood* **9,** 321–341 (2002).
22. Gardner, H. & Randall, D. The effects of the presence or absence of parents on interviews with children. *Nurse Res.* **19,** 6–10 (2012).

For peer review only

For peer review only - http://bmjopen.bmj.com/site/about/guidelines.xhtml

519

520 **Table 1: Demographic information of semi-structured interview participants**

	South Asian children (%)	White British children (%)
Total number	33	14
Male	20 (61)	8 (57)
Female	13 (39)	6 (43)
BTS grading		
Level 1	7 (21)	3 (21)
Level 2	17 (52)	8 (57)
Level 3	6 (18)	3 (21)
Level 4	3 (9)	0
Ethno-religious/ethnic group		
Indian Gujarati Hindu	6 (18)	-
Indian Gujarati Muslim	4 (12)	-
Indian Gujarati Jain	1 (3)	-
Indian Punjabi Sikh	6 (18)	-
Indian Punjabi Hindu	1 (3)	-

For peer review only

**Bangladeshi Muslim**	**9 (27)**	**-**
**Pakistani Muslim**	**6 (18)**	**-**
**White British**	**-**	**13 (93)**
**White British (English)**		**1 (7)**
**White British (Wales)**	**-**	**0**
**White British (Scottish)**	**-**	**0**
**White British (Northern Irish)**	**-**	**0**
**Religion**		
**Hindu**	**7 (21)**	**0**
**Muslim**	**19 (58)**	**0**
**Sikh**	**6 (18)**	**0**
**Jain**	**1 (3)**	**0**
**Christian**	**0**	**6 (43)**
**Atheist**	**0**	**1 (7)**
**No religious belief**	**0**	**4 (29)**
**Not specified**	**0**	**3 (21)**

For peer review only

**Age**		
**5 years old**	**2 (6)**	**2 (14)**
**6 years old**	**5 (15)**	**2 (14)**
**7 years old**	**5 (15)**	**0**
**8 years old**	**4 (12)**	**4 (29)**
**9 years old**	**3 (9)**	**1 (7)**
**10 years old**	**5 (15)**	**4 (29)**
**11 years old**	**5 (15)**	**1 (7)**
**12 years old**	**3 (9)**	**0**
**Not specified**	**1 (3)**	**0**
**Parental/ carer highest level of education achieved ***		
**Primary school**	**4 (8)**	**0**
**Secondary school**	**21 (43)**	**11 (37)**
**College education**	**3 (6)**	**3 (10)**
**University**	**20 (41)**	**14 (47)**
**Not specified**	**1 (2)**	**2 (7)**

521

For peer review only - http://bmjopen.bmj.com/site/about/guidelines.xhtml

*** Numbers may not be equal to the total number of children interviewed as either one or both parents responded to the questionnaire**

For peer review only

For peer review only - http://bmjopen.bmj.com/site/about/guidelines.xhtml

Supplementary file 1: Semi-structured interview question schedule

Question schedule for children

Hello. Thank you very much for agreeing to speak to me. So I'm (interviewer), and this is an interview for the MIA research study. We' re going to talk about your breathing. I'm going to ask you things like what you remember from when you were very little, and I'm going to ask you if you remember what it's like now. I'll also ask some questions about what happens when you have a bad day, medicines you take and what other people say about your asthma. If you don't know the answer, you can just guess. There's no right or wrong answer.

1. When did your breathing problems first start?
    - Initial symptoms/recognition
    - Who/how diagnosed
    - Information/support provided
    - Barriers to diagnosis
        - Seeking help
        - HCP attitude
        - Access to service
2. People have lots of different Ideas about why some children get wheezy. Why do you think experience wheeziness?
    - Triggers vs. cause
    - Heritability
    - Contagion
    - Gods will
    - Diet

For peer review only

3. What do you think causes asthma/ wheezing?
4. Could you describe what's your asthma like on a day-to-day basis
    - Symptoms
    - Impact on day-to-day life
    - Lifestyle restriction
    - Time off work
    - Family life
    - Schooling
5. Who makes the decision about day-to-day things?
    - Mum/dad
    - Child
    - Extended family
6. Could you describe a recent asthma attack? (substitute bad attack if little response)
    - Describe management
        - Use of health services
        - Use of tests/diagnostics
        - Role of extended family in management
7. Problems faces
    - Recognition of need to seek help
    - Access to service A&E/ walk in/ GP
    - Practicalities- carrying medicines, time of day, weekends etc
    - HCP communication/attitudes/ethnicity/ gender
8. What worked well?
    - Good service

For peer review only

- Good person

9. Information provided
    - What
    - From who
    - Useful/not
    - What else wanted
    - Timing
10. Solutions used/wanted
11. Let's talk about treatments. What do you hope treatments will do for your asthma/wheezing?
    - Personal goals of treatment
    - Management vs. cure
    - Concept of asthma control
    - What is good control
12. Do you take any medicines for your asthma/wheezing? (substitute inhalers if no response) (go through medicine one by one)
    - When do you use this/how do you feel about it/any problems getting taking it/ how do you remember to take it/ does everyone in the family know about it/ use in public?
    - For preventer medicines: when do you stop/ who makes decisions/ side effects?
    - Specific attitudes
        - Long term med use
        - Inhaler vs. tablets
        - Steroids

For peer review only

13. Who has helped you learn about wheezing/asthma?

- Sources of knowledge
- Skills
- Overseas advice
- Role of schools/community centres/religious centres
- Use of written management plans

14. What do the rest of the family think about your asthma?

- Who knows/ gets involved
- What do you think
- Does anyone offer advice/ do you follow it?
- Impact on management

15. What about your friends?

- Do you tell others/ stigma?
- Would you tell about another illness/specific to asthma?
- Common beliefs about cause
- Common attitudes towards asthma/ children with asthma
- Advice offered/ follow it
- Has anyone upset you?
- Do cultural attitudes influence management?

16. Where do you go for help about your asthma?

- Useful source of care- GP/A&E/walk-in/family and friends/internet/religious leaders
    - Who do you go to?
    - What is the key issue- opening hours, location, relationship, gender, ethnicity

For peer review only

- Problems faced getting help
  - Communication with HCPs
  - Getting prescriptions

17. What would overcome these?

18. Is there anything that you've found particularly helpful in taking care of your asthma?

- What/why?
- Alternative therapies used?
  - What/why
  - Diet
  - Heating/furnishing changes
  - Barriers or facilitator to using different options

19. Is there anything else we could do to help you cope with your asthma?

- What/why/how
  - Specific to your family
  - General suggestions for families and children

For peer review only

Consolidated criteria for reporting qualitative studies (COREQ): 32-item checklist[1]

No	Item	Guide questions/description	Checklist
**Domain 1: Research team and reflexivity**			
**Personal Characteristics**			
**1.**	Interviewer/facilitator	Which author/s conducted the interview or focus group?	Dr Deborah Bird Dr Melanie McFeeters
**2.**	Credentials	What were the researcher's credentials? *E.g. PhD, MD*	See 'authors and institution'; Line 4-72
**3.**	Occupation	What was their occupation at the time of the study?	See 'authors and institution'; Line 4-72
**4.**	Gender	Was the researcher male or female?	Female; Line 197
**5.**	Experience and training	What experience or training did the researcher have?	Page 10;Line 197-198
**Relationship with participants**			
**6.**	Relationship established	Was a relationship established prior to study commencement?	Yes; via encouraging children to draw and write about their asthma experience; Line 195-196
**7.**	Participant knowledge of the interviewer	What did the participants know about the researcher? e.g. *personal goals, reasons for doing the research*	Supplementary file 1
**8.**	Interviewer characteristics	What characteristics were reported about the interviewer/facilitator? e.g. *Bias, assumptions, reasons and interests in the research topic*	Line 197-198. Interviewer characteristics reported are: gender, profession, level

			of training in research and conflict of interests
		**Domain 2: study design**	
**Theoretical framework**			
**9.**	Methodological orientation and Theory	What methodological orientation was stated to underpin the study? *e.g. grounded theory, discourse analysis, ethnography, phenomenology, content analysis*	Semi-structured interview (grounded theory); Line 170
**Participant selection**			
**10.**	Sampling	How were participants selected? *e.g. purposive, convenience, consecutive, snowball*	Purposive sampling; Line 173-176
**11.**	Method of approach	How were participants approached? e.*g. face-to-face, telephone, mail, email*	Face-to-face and telephone; Line 173-176
**12.**	Sample size	How many participants were in the study?	Line 184
**13.**	Non-participation	How many people refused to participate or dropped out? Reasons?	Due to loss of interest, some children did not wish to participate in the interviews (n=2)
**Setting**			
**14.**	Setting of data collection	Where was the data collected? e.*g. home, clinic, workplace*	Family home; Line 198-199
**15.**	Presence of non-participants	Was anyone else present besides the participants and researchers?	Some children gave interviews independently, while some with their

			parents. Line 198-199
**16.**	Description of sample	What are the important characteristics of the sample? *e.g. demographic data, date*	Table 1. Line 573-576
**Data collection**			
**17.**	Interview guide	Were questions, prompts, guides provided by the authors? Was it pilot tested?	Supplementary file 1
**18.**	Repeat interviews	Were repeat interviews carried out? If yes, how many?	No repeat interviews were conducted. Line 200-201
**19.**	Audio/visual recording	Did the research use audio or visual recording to collect the data?	Interviews were digitally voice-recorded. Line 200
**20.**	Field notes	Were field notes made during and/or after the interview or focus group?	Fields notes were taken. Line 200
**21.**	Duration	What was the duration of the interviews or focus group?	Interviews typically lasted between 20-60 minutes [1] (see foot note)
**22.**	Data saturation	Was data saturation discussed?	No
**23.**	Transcripts returned	Were transcripts returned to participants for comment and/or correction?	Transcripts were not returned to children for comments/ corrections. Line 204-205

[1] Monica Lakhanpaul and others, *The Use of a Collaborative Structured Methodology for the Development of a Multifaceted Intervention Programme for the Management of Asthma (the MIA Project), Tailored to the Needs of Children and Families of South Asian Origin: A Community-Based, Partici* (NIHR Journals Library, 2014)

Domain 3: analysis and findings			
**Data analysis**			
**24.**	Number of data coders	How many data coders coded the data?	There were two data coders. Line 207
**25.**	Description of the coding tree	Did authors provide a description of the coding tree?	Line 206-212
**26.**	Derivation of themes	Were themes identified in advance or derived from the data?	Open coding with themes derived from the data; Line 204-212
**27.**	Software	What software, if applicable, was used to manage the data?	NVivo (QSR International, Warrington, UK); Line 204
**28.**	Participant checking	Did participants provide feedback on the findings?	Children took part in community dissemination events to provide feedback of the findings; Line 218-219
**Reporting**			
**29.**	Quotations presented	Were participant quotations presented to illustrate the themes / findings? Was each quotation identified? e.*g. participant number*	*Results;* Line 221-366
**30.**	Data and findings consistent	Was there consistency between the data presented and the findings?	*Discussion;* Line 374-425
**31.**	Clarity of major themes	Were major themes clearly presented in the findings?	*Discussion;* Line 374-425

**32.**	Clarity of minor themes	Is there a description of diverse cases or discussion of minor themes?	*Discussion;* Line 374-425

1. Allison Tong, Peter Sainsbury, Jonathan Craig; Consolidated criteria for reporting qualitative research (COREQ): a 32-item checklist for interviews and focus groups, *International Journal for Quality in Health Care*, Volume 19, Issue 6, 1 December 2007, Pages 349–357, https://doi.org/10.1093/intqhc/mzm042

For peer review only
